# Uncovering the Dynamic Alterations of Volatile Components in Sweet and Floral Aroma Black Tea during Processing

**DOI:** 10.3390/foods13050728

**Published:** 2024-02-28

**Authors:** Yanqin Yang, Qiwei Wang, Jialing Xie, Yuliang Deng, Jiayi Zhu, Zhongwen Xie, Haibo Yuan, Yongwen Jiang

**Affiliations:** 1 Key Laboratory of Biology, Genetics and Breeding of Special Economic Animals and Plants, Ministry of Agriculture and Rural Affairs, Tea Research Institute, Chinese Academy of Agricultural Sciences, Hangzhou 310008, China; yangyq@tricaas.com (Y.Y.);; 2 State Key Laboratory of Tea Plant Biology and Utilization, Anhui Agricultural University, 130 Changjiang West Road, Hefei 230036, China

**Keywords:** dynamic alterations, processing, GC-E-Nose, GC-IMS, GC-MS

## Abstract

Aroma is an indispensable factor that substantially impacts the quality assessment of black tea. This study aims to uncover the dynamic alterations in the sweet and floral aroma black tea (SFABT) throughout various manufacturing stages using a comprehensive analytical approach integrating gas chromatography electronic nose, gas chromatography–ion mobility spectrometry (GC-IMS), and gas chromatography–mass spectrometry (GC-MS). Notable alterations in volatile components were discerned during processing, predominantly during the rolling stage. A total of 59 typical volatile compounds were identified through GC-IMS, whereas 106 volatile components were recognized via GC-MS throughout the entire manufacturing process. Among them, 14 volatile compounds, such as linalool, *β*-ionone, dimethyl sulfide, and 1-octen-3-ol, stood out as characteristic components responsible for SFABT with relative odor activity values exceeding one. This study serves as an invaluable theoretical platform for strategic controllable processing of superior-quality black tea.

## 1. Introduction

Owing to its pleasant flavor and a multitude of health advantages, such as antioxidation, cancer-inhibiting, and antibacterial properties, black tea is the most extensively consumed and predominantly manufactured tea globally [1,2]. As a fully fermented tea, black tea is made through a sequence of procedures that include withering, rolling, fermentation, and drying [3]. Black tea is renowned for its distinctive flavor. Aroma is a critical indicator of the quality of black tea and largely determines consumer preference and approval. Tea aroma can vary due to different origins, varieties, and processing techniques [4,5]. The sweet and floral aroma black tea (SFABT) is representative of black tea favored by consumers for its harmonious and pleasant aroma. However, the dynamic change in SFABT during the manufacturing and processing is still unclear. A lack of knowledge of the volatile dynamic change restricts the quality control and processing of SFABT to a certain extent.

During the processing of tea, over 600 volatile substances have been identified [6]. A variety of analytical techniques have been used for tea flavor evaluation. Among these, gas chromatography–mass spectrometry (GC-MS) is the most commonly used technique to analyze flavor compounds [7,8,9,10]. Su et al. successfully used GC-MS to explore the relationship between the grade and the characteristic aroma in Keemun black tea [11]. Concurrently, there has been an increasing adoption of the electronic nose for flavor characterization [12]. However, traditional metal-oxide- or polymer-based electronic nose technologies are constrained by sensor drift and contamination, along with the limitations of the sensors [13]. In contrast, the gas-chromatography-based electronic nose (GC-E-Nose) offers a viable alternative. This technology combines sensor technology and fast gas chromatography separation to provide more molecular correlation. It is rapid, easy to use, reliable, and accurate [14]. Furthermore, gas chromatography–ion mobility spectrometry (GC-IMS) is endowed with the capability of separating and characterizing ionized molecules under ambient pressures and temperatures through their migration rates under an electrical field [15]. Considering its remarkable high sensitivity, ease of operation, and the potential for miniaturization, GC-IMS exhibits promise in its application in food flavor analysis, especially concerning trace volatile compounds [16,17,18,19,20].

The employment of various flavor analysis methodologies is crucial for delivering comprehensive, scientific, and compelling information. This paper investigates the volatile components at distinct stages of SFABT’s processing cycle via the utilization of SFABT using GC-E-Nose, GC-IMS, and GC-MS. In addition, the dynamic changes and key aroma-active compounds are effectively characterized by multivariate statistical analysis and relative odor activity value (rOAV) analysis. A potential formation mechanism is also discussed. This research aims to study the dynamic alterations of volatile components throughout the manufacturing process of black tea, which can aid in improving black tea quality.

## 2. Materials and Methods

### 2.1. Materials and Chemicals

The 20 mL headspace vials, accompanied by 18 mm magnetic PTFE/Silicone caps, were supplied by Agilent Technologies Inc. (Palo Alto, CA, USA). The Jinguanyin’ cultivar, selected for its strong, sweet, and floral aroma [21], was selectively harvested for its fresh leaves, ranging from one bud and one leaf to one bud and two leaves, in Shengzhou, Zhejiang Province during the initial period of April 2021. The information on the chemicals used in this study is listed in Table S1.

### 2.2. Preparation of Samples

Black tea processing involved the following stages: Initially, fresh leaves were exposed to spreading for 15 h at 27.6 °C, under a controlled air humidity level of 57%. This stage was crucial for attaining a moisture range of 60–64%. Subsequently, the tea leaves underwent shaking for 6 min at a speed of 30 rpm, followed by 10 min at a speed of 40 rpm. The time interval between the two shaking periods was 1 h. The tea leaves were rolled using a series of multi-step rolling procedures employing different pressures: no-pressure rolling (25 min), light-pressure rolling (20 min), heavy-pressure rolling (10 min), light-pressure rolling (15 min), and no-pressure rolling (5 min). Then, they underwent fermentation (at 28 °C for 2 h). The tea leaves were dried in a hot-air drying machine (6CHZ-7B type, Fujian Jiayou Machinery Co., Ltd., Quanzhou, China): first drying (at 110 °C for about 15 min until the water content had reached 18~20%, and then spread out for a further 30 min), and then final firing (at 85 °C for 35 min until the water content had reached ~5% moisture). The finished tea samples with typical sweet and floral aroma characteristics were stored at −20 °C for future analysis.

### 2.3. GC-E-Nose Analysis

The volatile profiles of SFABT during processing were analyzed utilizing the GC-E-Nose (Alpha M.O.S., Toulouse, France). Specifically, an accurately weighed tea sample (0.5 g) was transferred to a 20 mL sealed headspace vial with an incubation at 60 °C for a period of 20 min under a constant agitation speed of 500 rpm [22]. A volume of 5000 μL of headspace gas with a rate of 300 μL/s was introduced into the system. The volatile components were trapped on a Tenax TA trap at a temperature of 20 °C for a duration of 27 s, followed by a thermal desorption protocol conducted at 240 °C for a span of 30 s. For the separation of volatiles, two chromatography columns with disparate polarities were utilized. The columns included the MXT-5 and MXT-1701 (both measuring 20 m × 0.18 mm I.D. × 0.4 μm, manufactured by Restek, Bellefonte, PA, USA). An initial oven temperature of 50 °C was maintained for 5 s before elevating it at a rate of 0.1 °C/s up to 80 °C, and ultimately, raised further to 250 °C at a rate of 0.4 °C/s, maintaining this temperature for 10 additional seconds. The entire temperature profiling duration was 740 s. Both flame ionization detectors were adjusted to a temperature of 260 °C. Each sample was subjected to analysis in triplicate.

### 2.4. GC-IMS Analysis

The volatile fingerprints at different manufacturing stages of SFABT were executed using a state-of-the-art GC-IMS apparatus (Flavourspec^®^, G.A.S, Dortmund, Germany). The analysis conditions referred to our previous studies [23]. Briefly, tea samples (1.0 g) were loaded into a 20 mL headspace vial. The samples were subsequently subjected to incubation at a consistent temperature of 60 °C for a duration of 15 min, with a constant agitation speed of 500 rpm. Post-incubation, 500 μL of the headspace samples was transferred to the injection port through a preheated syringe maintained at 85 °C. Volatile constituents were isolated utilizing an MXT-5 capillary column (15 m × 0.53 mm × 1 μm; Restek, Beijing, China). High-purity nitrogen (99.999%) served as the carrier gas with a programmed procedure: 0~2 min, 2 mL/min, and 2~20 min, 100 mL/min. The flow velocity of the drift gas (nitrogen) within the drift tube was adjusted to 150 mL/min. The column temperature was regulated at 60 °C, while the drift tube temperature was kept at 45 °C. All analyses were performed in triplicate.

The retention index (RI) of the volatile compounds was determined utilizing *n*-ketones C4-C9 (Sinopharm Chemical Reagent Beijing Co., Ltd., Beijing, China). The volatile compounds were qualitatively evaluated through a comparative analysis of RI values and the drift time against the GC-IMS library. The obtained IMS data were analyzed by using built-in software, such as VOCal 0.4.03, which was used to visually display the analytical spectrum. The Reporter plug-in 1.4.00 functioned to generate two-dimensional and three-dimensional diagrams, respectively. The Gallery Plot plug-in 1.2.8 performed visual fingerprint comparisons between different samples.

### 2.5. GC-MS Analysis

The volatile components were carried out using an 7890B/7000C GC-MS analytical instrument (Agilent Technologies, Palo Alto, CA, USA). In brief, homogenized tea samples (0.5 g) were transferred into a 20 mL headspace vial. Subsequently, 6 mL of boiling water along with 5 μL of ethyl decanoate (with a concentration of 100 mg/L serving as the internal standard) were added, strictly adhering to our prior methodology [23]. The headspace vial was immediately sealed with a screw cap. The samples were incubated using metal-bathing at 60 °C, and a manual DVB/CAR/PDMS fiber (50/30 μm, Supelco, Bellefonte, PA, USA) was subsequently introduced into the aforementioned headspace vial and subjected to exposure for a period of 60 min. Post-extraction, the DVB/CAR/PDMS fiber was transferred directly into the injector port and subjected to thermal desorption (lasting 5 min) at a temperature of 250 °C. The HP-5ms capillary column, manufactured by Agilent Technologies Inc., was employed to facilitate the separation of volatile compounds, with dimensions of 60 m × 250 μm × 0.25 μm. Highly purified helium (99.9995%) was employed as the carrier gas under a consistent flow rate of 1 mL/min. The split-less mode was implemented. The programmed temperature profile was set as follows: beginning at 40 °C for 5 min, steadily increasing to 100 °C at a rate of 6 °C/min (holding for 2 min), and finally, ramping up to 270 °C at a rate of 5 °C/min (holding for 4 min). The mass spectrometer was operational in the mode of electron ionization (EI), exhibiting a scanning extension from 33 to 550 *m*/*z*. The ion source temperature and transfer line temperature were set at 230 °C and 270 °C, respectively.

The qualitative assessment was performed on the MassHunter Workstation Software Unknowns Analysis B.07.01 platform for insightful interpretation. The compounds were identified through meticulous comparison of acquired spectra against those from the prestigious National Institute of Standards and Technology (NIST11) and well-established retention indices derived through the linear calculation formula of *n*-alkanes (C7–C40). The volatile compounds were semi-quantitatively analyzed by equating the peak intensities of each volatile to that of the internal standard (ethyl decanoate). Each sample underwent triplicate analysis.

### 2.6. rOAV Analysis

rOAV was determined utilizing the following formula:

rOAV = C/OT

In this equation, ‘C’ signifies the content of a particular volatile compound, while ‘OT’ denotes its odor threshold. 

### 2.7. Scanning Electron Microscopy

Images were captured utilizing the HITACHI SU-8010 high-resolution field-emission scanning electron microscope. The specific tea samples were affixed to the sample platform through the assistance of conductive carbon glue and, subsequently, gold was adorned using an ionic sputtering apparatus for approximately 30 s, culminating in inspection under a scanning electron microscope for data acquisition and evaluation.

### 2.8. Statistical Analysis

Both partial least squares discriminant analysis (PLS-DA) and orthogonal PLS-DA (OPLS-DA) were successfully implemented by employing the SIMCA-P 13.0 software (Umetrics, Umea, Sweden). Multiple comparisons were performed utilizing one-way analysis of variance (ANOVA), employing SPSS statistics 20.0 (SPSS Inc., Chicago, IL, USA). The column was accomplished via the utilization of Origin 9.1 software (OriginLab Corporation, Northampton, MA, USA). Data visualization was facilitated through the creation of heat maps using MultiExperiment Viewer 4.9.0 (Oracle Corporation, Redwood Shores, CA, USA).

## 3. Results and Discussion

### 3.1. Dynamic Alterations during Processing Analyzed via GC-E-Nose

As a pioneering electronic sensing methodology, the GC-E-Nose is capable of executing real-time online surveillance of volatile fingerprint changes. The typical logarithmic radar diagrams of SFABT within the final firing processing acquired from the MXT-5 and MXT-1701 columns are shown in Figure S1. Each peak corresponds to a specific volatile compound, with the peak area correlated with the volatile content.

**Figure 1:**
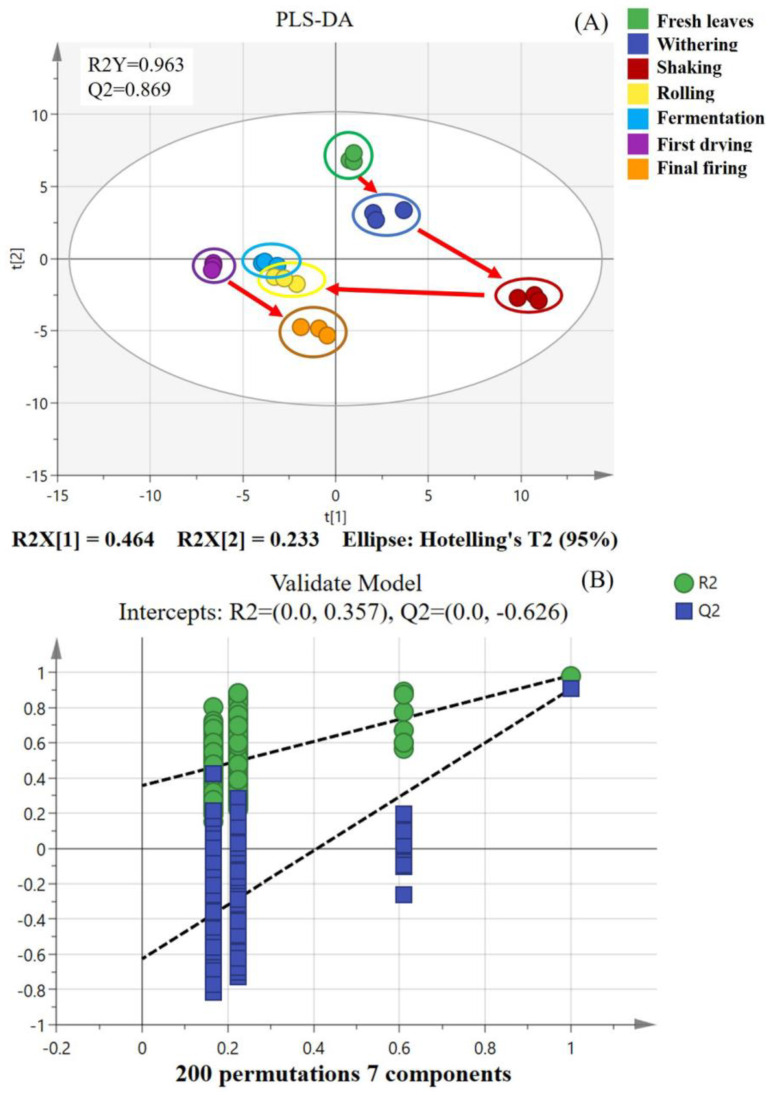
PLS-DA of SFABT over the entire manufacturing process via the GC-E-Nose. (**A**) Scores of PLS-DA (R^2^Y = 0.963 and Q^2^ = 0.869). (**B**) Cross-validation by a 200-fold permutation test (R^2^ = 0.357 and Q^2^ = −0.626).

To gain insight into the dynamic alterations in volatile fingerprints of SFABT throughout the manufacturing process, PLS-DA was employed as an effective multivariate statistical technique. As a supervised classification analysis method, PLS-DA possesses the capability to distinguish observations across groups and can pinpoint the influential variables contributing to the disparities by appropriately rotating the principal components. The model parameters (R^2^Y = 0.963, Q^2^ = 0.869) suggested an excellent fit with high predictive power (Figure 1A). To scrutinize the robustness of the model, a total of 200 iterations of the permutation test were carried out. No over-fitting phenomena were observed, with the model parameters as follows: R^2^ = 0.357 and Q^2^ = −0.626 (Figure 1B). The PLS-DA score shows that the volatile fingerprints of SFABT were in constant dynamic change during the whole process. Notably, considerable fluctuations emerged in volatiles within the stages of shaking, rolling, and final firing, primarily during the processes of shaking and rolling. Wang et al. addressed the effects of shaking on the evolutionary mechanisms of volatile metabolites of black tea and found that shaking could stimulate the oxidative degradation of fatty acids and carotenoids to promote the formation of floral/fruity volatile metabolites [24]. Rolling, a critical step in the formation of SFABT, destroys leaf cells and ensures the uniform maceration of tea leaves, prompting aroma precursors to precipitate into the cells. These substances subsequently endure enzymatic oxidation, yielding the generation of numerous volatile substances [23,25,26]. In addition, scanning electron microscopy was implemented to further observe the mechanism of dynamic change in the microstructure. The results show that the leaf epidermis gradually appeared to fold after the withering and shaking processes. The leaf tissue was seriously damaged during the rolling stage (Figure S2), which further confirms the above phenomenon. In addition, Wu et al. (2019) [27] investigated the dynamic alterations of chemical compositions in black tea during processing and discovered that the most notable disparities occurred in the withering and rolling stages, which is consistent with our results. In conclusion, the results show that the fingerprint changes of SFABT could be quickly and effectively characterized by the GC-E-Nose.

### 3.2. Dynamic Changes during Processing Analyzed via GC-IMS

#### 3.2.1. GC-IMS Topographic Plots in Different Processing Stages of SFABT

**Figure 2:**
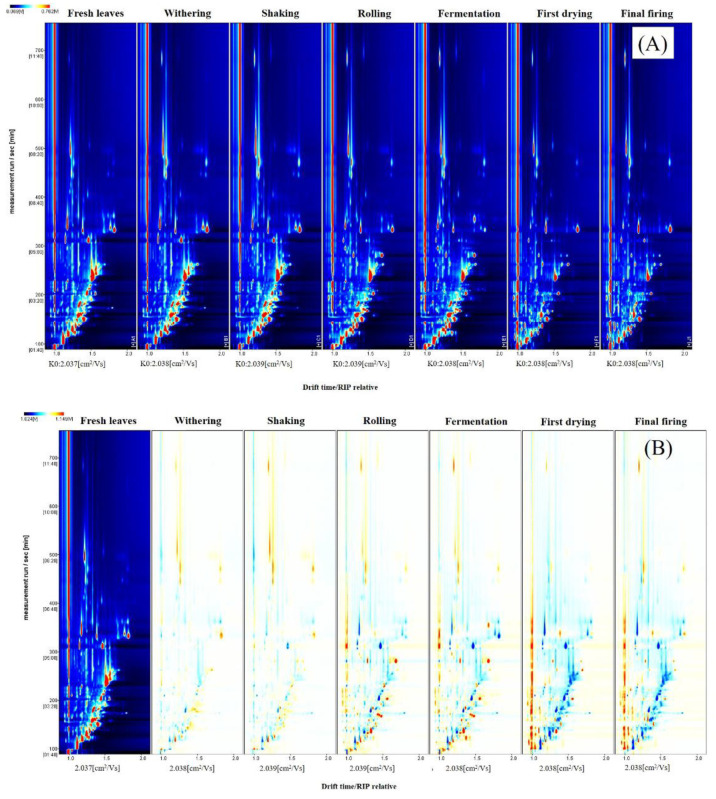
Fingerprints of SFABT during the entire manufacturing process obtained from GC-IMS: (**A**) two-dimensional topographic plot and (**B**) difference comparison plots.

In order to facilitate the identification of volatile components and their variation regularity in SFABT during the manufacturing process, the technique known as GC-IMS was employed to extract comprehensive IMS insights from the examined samples. The volatile components in SFABT are shown via topographic plots (Figure 2A), with the ordinate signifying the retention time, and the abscissa capturing the ion migration time [15]. A red vertical line positioned at abscissa 1.0 represents the reactive ion peak (RIP), which has been pre-normalized. The background of each topographical profile is rendered in pristine blue, with the color signifying the intensity levels of the volatile compounds. Specifically, shades of red designate higher concentrations, while white demonstrates diminished levels. The deeper the shade, the higher the signal intensity. To facilitate visualization and comparative analysis, a spectral diagram obtained from fresh leaves was chosen as the benchmark, and the corresponding spectrum displayed by processed tea samples was deducted from the referential data. If a specific volatile compound remained consistent, the post-deduction background would appear white. The presence of red and blue implies that the concentration of certain volatile components was either heightened or reduced relative to the reference sample. Most of the signals were distributed in a retention time of 100–400 s and a drift time of 1.0–1.8. It is evident from Figure 2B that certain compounds diminished in SFABT as the processing phases progressed, and novel substances emerged, likely owing to the intricate biological and chemical mechanisms inherent in the fabrication process [6].

#### 3.2.2. Qualitative Results of Volatile Components during SFABT Processing 

In this study, a comprehensive analysis unearthed a total of 100 signal peaks, out of which 59 recognizable typical target volatiles emerged (Table S2). The predominant compounds within these groupings included 11 alcohols, 1 sulfide, 13 aldehydes, 3 terpenes, 12 ketones, 6 heterocycles, and 13 esters. Nevertheless, there remained 23 peaks that failed to receive identification. Certain individual compounds may generate multiple signals or spots (such as dimers and even trimers), which could potentially be ascribed to their different contents [28]. Notably, the drift time of dimers was greater because of their proton affinities and elevated contents. 

**Figure 3:**
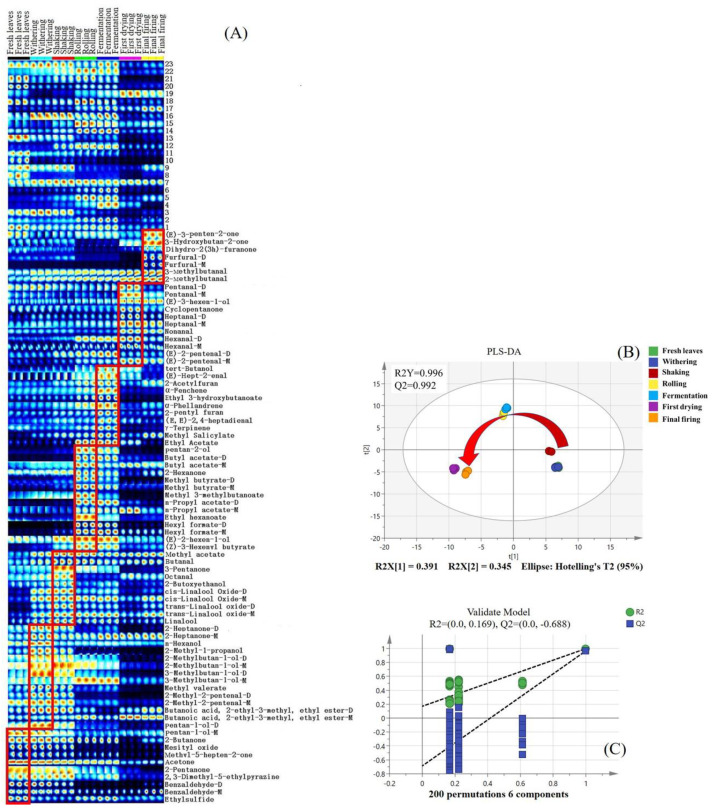
Fingerprints of SFABT during the entire manufacturing process obtained from GC-IMS. (**A**) Fingerprints of all the samples. The suffix -M represents a monomer of volatile components, while the suffix -D represents a dimer. (**B**) Scores of PLS-DA with R^2^Y = 0.996 and Q^2^ = 0.992. (**C**) Cross-validation through a 200-fold permutation examination, yielding an R^2^ score of 0.169 and Q^2^ at −0.688.

Although the topographic plots showed tendencies of the volatile compounds, accurate judgment regarding specific components was challenging. The fingerprint is an effective way of addressing this problem, where each row depicts the complete signal peak of a single sample, and each column denotes the identical components across disparate samples. The content is signified by the color code of the square—the brighter the color, the higher the content. Known components are denoted with their existing names, whereas unidentified components are labeled with numbers. As depicted in Figure 3A, there was an appreciable enrichment in the contents of pentan-1-ol monomer (#68), benzaldehyde dimer (#21), and ethylsulfide (#80) in fresh leaves, as compared to their concentrations in the remaining processed stages. In the withering stage, the concentration levels of 2-heptanone (#31, #35), 2-methyl-1-propanol (#82), *n*-hexanol (#97), 2-methylbutan-1-ol monomer (#92), 3-methylbutan-1-ol dimer (#79), methyl valerate (#77), 2-methyl-2-pentenal (#76, #81), and butanoic acid, 2-ethyl-3-methyl, ethyl ester dimer (#15) exceeded those in the other stages. The contents of methyl acetate (#66), butanal (#64), 3-pentanone (#83), octanal (#42), 2-butoxyethanol (#32), *cis*-linalool oxide dimer (#8), and *trans*-linalool oxide (#4, #5) were clearly enhanced in the shaking stage, albeit being relatively lower in the initial two stages. Their contents tended to decrease in subsequent stages. Concerning rolling, esters such as butyl acetate (#89, #100), methyl butyrate (#84, #88), methyl 3-methylbutanoate (#87), ethyl hexanoate (#44), and hexyl formate dimer (#24) were observed to be elevated in the rolling stage compared to the other processed samples. Significantly elevated levels of tert-butanol (#96), (*E*)-hept-2-enal (#33), 2-acetylfuran (#29), *α*-fenchene (#27), ethyl 3-hydroxybutanoate (#22), *α*-phellandrene (#19), 2-pentyl furan (#17), (*E, E*)-2,4-heptadienal (#12), *γ*-terpinene (#9), and methyl salicylate (#2) were observed during the fermentation process. Regarding the first drying stage, aldehyde compounds such as pentanal (#57, #58), heptanal (#25, #26), nonanal (#45), hexanal (#49, #50), and (*E*)-2-pentenal (#52, #53) were found to be more abundant in the first drying stage than in the other processed samples. It is hypothesized that the heating process could potentially enhance the degradation of fatty acids, subsequently generating the associated fatty aldehyde. For the final firing process, the contents of (*E*)-3-penten-2-one (#73), 3-hydroxybutan-2-one (#71), dihydro-2(3h)-furanone (#39), furfural (#47, #48), 2-methylbutanal (#59), and 3-methylbutanal (#60) significantly exceeded those observed at preceding stages. Principle compounds, including dihydro-2(3H)-furanone and furfural, are potentially derived from the associated Maillard reaction during the thermal process [29].

#### 3.2.3. Multivariate Statistical Analysis

In order to more effectively delineate and distinguish the variations among the black tea samples throughout all the processing stages, PLS-DA was implemented utilizing the volatile components identified through GC-IMS. A satisfactory result was obtained with the model parameters of R^2^Y = 0.996 and Q^2^ = 0.992, respectively. Remarkably, the seven stages of black tea processing could be distinctly distinguished from each other, particularly between shaking and rolling, and between fermentation and first drying (see Figure 3B). These results suggested that the most striking changes in the volatile components occurred during the rolling and first-drying stages, compared with the other processing stages. An in-depth study of the model was conducted via a comprehensive permutation test encompassing 200 iterations, and the parameters (R^2^ = 0.169 and Q^2^ = −0.688) indicated a strong robustness of the model (Figure 3C). The above results indicated that the volatile metabolites changed greatly after rolling processing, which were in line with the results of PLS-DA on volatile fingerprints analyzed via the GC-E-Nose.

### 3.3. Dynamic Changes during Processing Analyzed via GC-MS

#### 3.3.1. Analysis of Volatile Compounds in SFABT across All the Processing Steps

**Figure 4:**
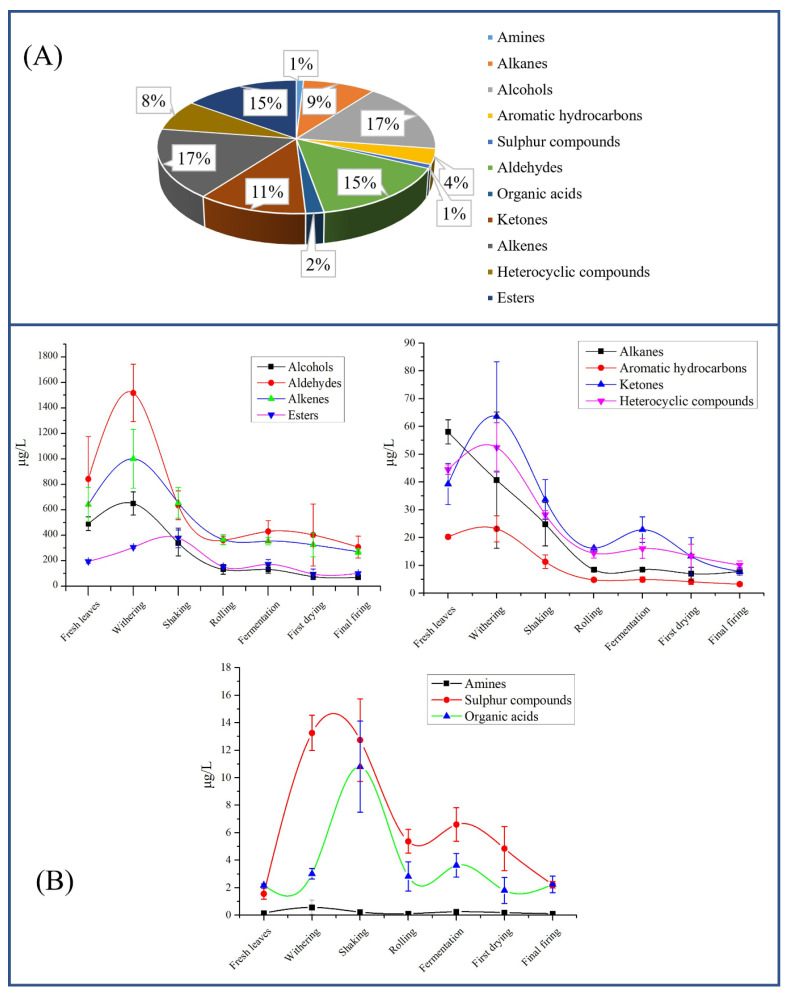
The volatiles identified throughout the entirety of the manufacturing process for SFABT via GC-MS. (**A**) Comparative proportions of volatiles across distinct categories. (**B**) Dynamic alterations of various volatile classifications throughout the complete manufacturing process.

A comprehensive analysis of volatile components during SFABT processing was performed by GC-MS. A total of 106 volatile compounds were identified, which comprised 11 categories (Table S3). Notably, the four component types accounting for the greatest relative proportion included alcohols (17%), alkenes (17%), aldehydes (15%), and esters (15%), as illustrated in Figure 4A. These volatile compounds exhibited significant variations across various processing stages (Figure 4B).

In terms of aldehydes, the highest content was observed during withering (1517.23 μg/L), followed by fresh leaves (841.45 μg/L), shaking (636.05 μg/L), fermentation (430.44 μg/L), first drying (402.30 μg/L), rolling (361.29 μg/L), and final firing (306.74 μg/L). The levels of alkenes, alcohols, ketones, heterocyclic compounds, and aromatic hydrocarbons exhibited analogous alterations. The content of esters exhibited an ascending trajectory from fresh leaves (194.64 μg/L) up to shaking (378.28 μg/L), and then showed a downward trend. The change trend of organic acids was the same as that of esters. The content of alkanes displayed a precipitous decrease from fresh leaves (58.02 μg/L) to rolling (8.44 μg/L), remaining constant in the subsequent stages. Sulfur compounds demonstrated an impressive augmentation by 8.5-fold (from 1.56 µg/L in fresh leaves to 13.26 µg/L in withering stage), which remained steady throughout the shaking stage. As the processing progressed, the level of sulfur compound drastically declined during the rolling stage (down to 5.37 μg/L), but subsequently, the content remained stable. We noted that the contents of amines displayed little variation throughout the entire process. Volatile constituents in the different categories showed different trends of change, potentially attributed to the release of volatiles arising from glycoside hydrolysis, carotenoid or lipid degradation, and the Maillard reaction during the manufacturing process [6,30].

#### 3.3.2. Dynamic Changes in SFABT at Different Manufacturing Stages

**Figure 5:**
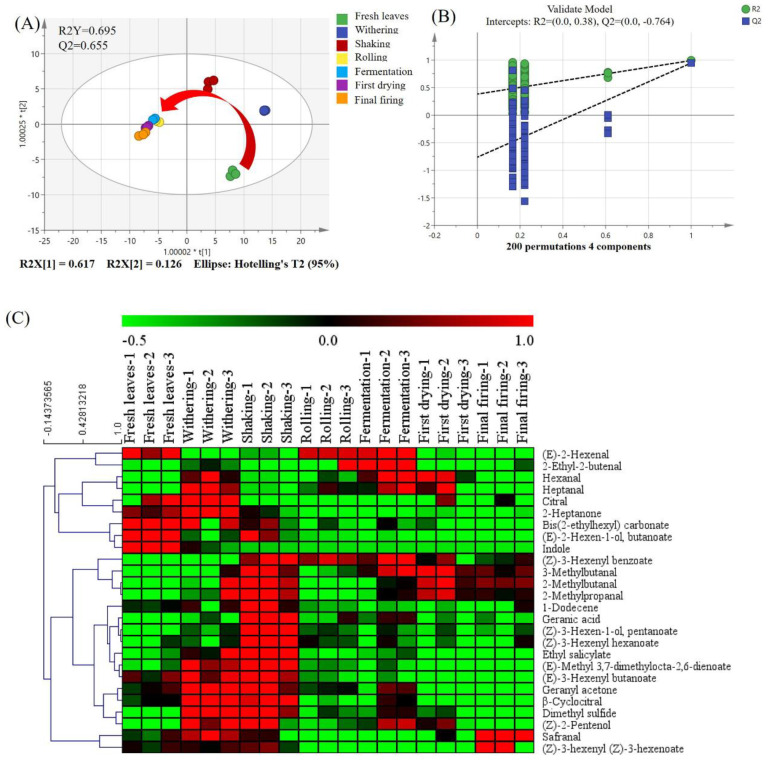
The results of multivariate statistical analysis obtained from GC-MS. (**A**) Scores of OPLS-DA (R^2^Y = 0.695 and Q^2^ = 0.655). (**B**) Cross-validation through a 200-fold permutation examination, yielding an R^2^ score of 0.38 and Q^2^ at −0.764. (**C**) Heat map analysis.

In order to more thoroughly comprehend the differences in the volatile profile of SFABT at different manufacturing stages, a total of 106 volatile compounds underwent OPLS-DA for assessing their variability. The model’s fitness was interpreted as excellent due to the high values of R^2^Y and Q^2^ achieved (Figure 5A). The score exhibited a distinct spatial segregation of SFABT during processing. Specifically, fresh leaves were situated within the fourth quadrant, while withered and shaken samples were stationed within the first quadrant. Rolled, fermented, and dried samples were positioned in the third quadrant. The closer the spatial distance was, the less the volatile metabolites changed; thus, noticeable alterations occurred in the rolling stage, which was consistent with the results of the GC-E-Nose and GC-IMS. A permutation test with a random 200-fold arrangement was executed to confirm the dependability of the OPLS-DA model. The results indicated that the OPLS-DA model was dependable and displayed no overfitting phenomena, as evidenced by the intercepts of R^2^ = (0.0, 0.38) and Q^2^ = (0.0, −0.764) (Figure 5B).

In addition, a dual criterion was implemented to determine the most prominent variables during processing. A total of 26 volatile biomarkers were pinpointed, strictly adhering to variable importance in the projection (VIP) exceeding 1.0 and the significance analysis (*p* < 0.05). An informative heat map was implemented to visually present the notable variations throughout the seven stages of black tea processing. Clear differences can be observed for these 26 volatile compounds, where green signifies reduced levels, while red denotes elevated levels. As shown in Figure 5C, indole (84) and (*E*)-2-hexenyl butanoate (67) showed higher concentrations in fresh leaves than in other manufacturing procedures. Heptanal (21), citral (81), 2-heptanone (17), dimethyl sulfide (1), and geranyl acetone (96) showed higher concentrations in the withering stage. As for the shaking processing, the representative compounds mainly included 2-methylpropanal (2), 2-methylbutanal (5), (*Z*)-3-hexenyl pentanoate (76), ethyl salicylate (82), and (*Z*)-3-hexenyl benzoate (103). As far as fermentation processing is concerned, higher concentrations of 2-ethyl-2-butenal (12) and (*E*)-2-hexenal (13) were found. Safranal (72) and (*Z*)-3-hexenyl (*Z*)-3-hexenoate (91) were found in higher concentrations in the final firing than in the other manufacturing procedures. The specific reasons for the fluctuations within the aforementioned volatiles will be investigated in depth in the future.

#### 3.3.3. rOAV Analysis of SFABT during the Manufacturing Process

The aroma profiles of tea are contingent upon the comprehensive performance of a certain proportion of aroma components, and the contribution of the detected odorants to the overall aroma is mainly dependent on their concentration level and documented odor threshold. Typically, volatile compounds possessing an rOAV ≥ 1 are deemed to contribute significantly to the characteristic aroma. In this study, the threshold values, odor descriptions, and calculated rOAVs of volatile components during SFABT processes are listed in Table 1. A total of 22 volatiles displayed rOAVs exceeding 1 in SFABT during the manufacturing process (Table S4). Of these, dimethyl sulfide (rOAV = 5.20~44.19), 1-octen-3-ol (rOAV = 1.28~12.51), *β*-myrcene (rOAV = 1.87~8.73), *β*-ocimene (rOAV = 455.01~2020.19), 3,5-octadien-2-one (rOAV = 3.63~42.60), linalool (rOAV = 145.13~958.49), methyl salicylate (rOAV = 1.34~5.52), *β*-cyclocitral (rOAV = 9.58~41.91), *β*-citral (rOAV = 3.53~11.46), and *β*-ionone (rOAV = 379.56~1759.80) showed rOAVs greater than 1 in each processing stage. Here, 3-methylbutanal (rOAV = 4.93~9.30) and 2-methylbutanal (rOAV = 2.06~5.93) exhibited OAVs greater than 1 in all the processes, except for in fresh leaves. Further, 2-heptanone (rOAV = 1.01~2.09) and *o*-cymene (rOAV = 1.29~1.43) displayed rOAVs exceeding 1 exclusively in fresh leaves and the withering stage, while decanal (rOAV = 2.66~1.22) and 2-ethylfuran (rOAV = 2.65~1.12) exhibited rOAVs surpassing 1 from fresh leaves to the shaking stage. (*E*)-2-Hexenal and 1-hexanol demonstrated rOAVs exceeding 1 solely in fermentation (rOAV = 1.15) and fresh leaves (rOAV = 1.22), respectively. A total of 14 volatile compounds, including dimethyl sulfide, methyl salicylate, *β*-cyclocitral, 2-methylpropanal, 3-methylbutanal, 2-methylbutanal, *β*-citral, citral, 1-octen-3-ol, *β*-myrcene, *β*-ocimene, 3,5-octadien-2-one, *β*-ionone, and linalool, were screened as the key constituents contributing to the quality of the finished SFABT. In addition, it was observed that the levels of certain volatile compounds may fall below the threshold value (rOAV < 1), but their potential influence on the aroma profile of SFABT should not be overlooked due to their potential additional effects, such as synergistic or masking effects.

#### 3.3.4. Overview of Related Mechanisms of Key Aroma-Active Compounds in SFABT

**Figure 6:**
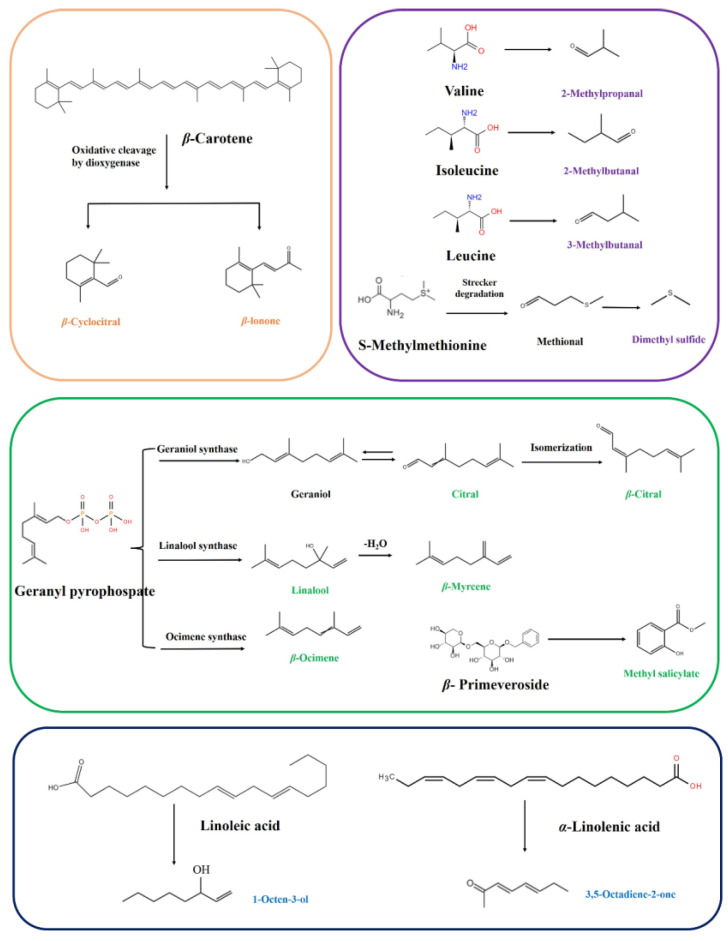
Possible formation mechanisms of key odorants in SFABT.

In exploring the classic metabolic pathways of volatiles found within tea, the mechanisms of degradation or transformation for the aforementioned 14 key aroma-active compounds were elucidated. Depending on the classes of volatile metabolites, they can be organized distinctly into four categories: two carotenoid-derived volatiles (CDVs), four amino-acid-derived volatiles (AADVs), six glycoside-derived volatiles (GDVs), and two fatty-acid-derived volatiles (FADVs) (Figure 6). Interestingly, the GDVs emerged as the dominant category, indicating that glycoside hydrolysis was the critical pathway for SFABT.

Within this array of pathways, *β*-cyclocitral and *β*-ionone were pivotal odorants derived from *β*-carotene degradation, which underwent enzymatic catalyzation by dioxygenases over the course of the fermentation process [6]. These CDVs predominantly impart unique floral and fruity odors. 

AADVs, including dimethyl sulfide, 2-methylpropanal, 2-methylbutanal, and 3-methylbutanal, predominantly occur through the Maillard reaction during thermal processing. Specifically, 2-methylpropanal, 2-methylbutanal, and 3-methylbutanal, categorized as Strecker aldehydes, were derived from their corresponding precursors: valine, isoleucine, and leucine, respectively [6]. Additionally, 2-methylpropanal, 3-methylbutanal, and 2-methylbutanal were reported to contribute to the malty flavors. Dimethyl sulfide was recognized for its cooked corn-like aroma. The potential formation mechanism suggests that amino acid of *S*-methylmethionine undergoes Strecker degradation to form dimethyl sulfide [31].

It has been demonstrated that linalool and *β*-ocimene, renowned for imparting floral and fruity scents in black tea, are derived from the primary geranyl pyrophosphate (geranyl-PP) substrate activated by linalool synthase and ocimene synthase, respectively [23]. Notably, linalool is subsequently dehydrated to form *β*-myrcene. Citral and *β*-citral are enantiomers of each other, primarily derived from geranyl-PP catalyzed by geraniol synthase. Methyl salicylate, known for its minty, fresh, and sweet fragrances, is liberated from *β*-primeveroside. 

The 1-octen-3-ol and 3,5-octadiene-2-one are typical FADVs. In cucumber, earth, fatty, and mushroom-like aromas, 1-octen-3-ol exerts a pivotal role, and has been documented to originate from linoleic acid, which is oxygenated by lipoxygenase (LOX) to form lipid hydroperoxides [30]. The 3,5-octadiene-2-one is associated with creamy and fruity flavors and is generated from *α*-linolenic acid. In the future, the formation pathway and mechanism will likely be traced in depth based on stable-isotope-labeling technology. The results will provide theoretical support for the directional regulation of SFABT.

## 4. Conclusions

Aroma presents as a crucial quality index of SFABT. Clarifying its key aroma-active compounds and their dynamic changes during processing holds substantial importance for the quality assurance of black tea. In this study, the integration of the GC-E-Nose, GC-IMS, and GC-MS effectively characterized the dynamic changes in SFABT during different manufacturing stages. The application of these three methodologies revealed pronounced alterations in volatile metabolites across the various stages of processing, specifically during the rolling step. A comprehensive analysis of 59 typical volatile compounds was accomplished via GC-IMS, whereas 106 volatile components were identified by GC-MS throughout the entire manufacturing process. Among them, 14 volatile compounds were screened as characteristic components responsible for SFABT with rOAVs exceeding one. Moreover, the related mechanisms of the key volatile compounds were also explored, and the GDVs emerged as the dominant category, indicating that glycoside hydrolysis was a pivotal pathway for the generation of characteristic aromas of SFABT. To a certain extent, this study has provided comprehensive insight into the dynamic alterations of SFABT during processing, thereby laying solid theoretical groundwork for strategic processing of SFABT. In addition, a novel quality control method of GC-IMS was incorporated into tea processing, holding immense potential for real-time monitoring of tea production regulation compared to traditional analytical techniques.

## Figures and Tables

**Table 1:** Odor descriptions and odor threshold values of the identified volatile components in SFABT during the entire manufacturing process.

No.	Compounds	Threshold Values (μg/L)	Literature Sources	Odor Descriptions	rOAVs						
					Fresh Leaves	Withering	Shaking	Rolling	Fermentation	First Drying	Final Firing
1	Dimethyl sulfide	0.3	d	Cooked corn-like	5.20	44.19	42.47	17.91	21.99	16.16	7.40
2	2-Methylpropanal	0.49	d	Malty	0.16	1.55	2.51	0.89	1.30	1.84	1.46
3	Acetic acid	200,000	e	Sour, pungent, vinegar	0.00	0.00	0.00	0.00	0.00	0.00	0.00
4	3-Methylbutanal	0.2	b	Malty	0.58	4.93	9.30	5.38	8.95	9.20	7.43
5	2-Methylbutanal	1	b	Malty	0.25	3.43	5.93	2.06	3.06	4.81	4.34
6	2-Ethylfuran	2.3	b	Grassy	2.65	1.95	1.12	0.46	0.52	0.60	0.42
7	2-Methylbutanol	211,800	k	Ethereal	0.00	0.00	0.00	0.00	0.00	0.00	0.00
8	3-Penten-2-one	1.5	h	Fruity smell	0.26	0.25	0.11	0.05	0.07	0.08	0.04
9	Toluene	527	a	Sweet, aromatic	0.00	0.00	0.00	0.00	0.00	0.00	0.00
10	(*Z*)-2-Pentenol	720	c	Green, fruity	0.00	0.00	0.00	0.00	0.00	0.00	0.00
11	Hexanal	4.5	c	Grassy, green, tallowy	0.55	1.77	0.81	0.91	1.77	1.52	0.51
12	2-Ethyl-2-butenal	n.f.		-	-	-	-	-	-	-	-
13	(*E*)-2-Hexenal	17	c	Green, fresh, fruity	0.97	0.26	0.47	0.95	1.15	0.41	0.23
14	(*E*)-3-Hexen-1-ol	70	a	Green, leafy, grassy	0.62	0.46	0.37	0.18	0.17	0.12	0.09
15	1-Hexanol	5.6	c	Green, cut grass	1.22	0.91	0.71	0.34	0.32	0.23	0.17
16	1,3-Dimethylbenzene(m-xylene)	1000	a	Plastic, green, pungent	0.00	0.00	0.00	0.00	0.00	0.00	0.00
17	2-Heptanone	1	f	Stale, cabbage-like	1.01	2.09	0.61	0.18	0.26	0.36	0.36
18	1,3,5,7-Cyclooctatetraene	n.f.		-	-	-	-	-	-	-	-
19	2-Heptanol	70	h	Fresh lemon, grass, herbal, sweet, floral, fruity, green	0.02	0.07	0.01	0.00	0.01	0.01	0.00
20	5-Hexen-3-one	n.f.		-	-	-	-	-	-	-	-
21	Heptanal	3	g	Heavy, plant-like green odor, apricot-like, and nutty aroma	0.23	0.62	0.25	0.35	0.45	0.39	0.15
22	Benzaldehyde	350	a	Almond-like, fruity, cherry-like, powdery, nutty	0.20	0.20	0.08	0.02	0.03	0.02	0.02
23	1-Heptanol	425	c	Leafy green, with vegetative and fruity odor	0.01	0.01	0.00	0.00	0.00	0.00	0.00
24	1-Octen-3-ol	1	c	Cucumber, earth, fatty, mushroom-like	7.36	12.51	4.60	1.93	2.31	1.96	1.28
25	6-Methyl-5-hepten-2-one	50	c	Fruity, apple-like, musty	0.04	0.06	0.02	0.01	0.01	0.00	0.00
26	(*E*)-2-(1-Pentenyl)furan	n.f.		Roasted	-	-	-	-	-	-	-
27	*α*-Phellandrene	36	c	Turpentine, minty	2.45	3.63	2.03	1.01	1.07	0.86	0.78
28	*β*-Myrcene	15	a	Woody, resinous, musty	5.88	8.73	4.88	2.43	2.57	2.05	1.87
29	2,2,4,6,6-Pentamethylheptane	n.f.		-	-	-	-	-	-	-	-
30	Decane	10,000	c	Alkane-like	0.00	0.00	0.00	0.00	0.00	0.00	0.00
31	Octanal	0.7	g	Pungent, fruity, and floral odor	0.79	0.91	0.35	0.25	0.29	0.32	0.20
32	*δ*-Carene	n.f.		-	-	-	-	-	-	-	-
33	2-Ethyl-1-hexanol	270	h	Citrus, fresh, floral, oily, sweet	0.00	0.01	0.00	0.00	0.00	0.00	0.00
34	*o*-Cymene	11.4	c	Aromatic	1.29	1.43	0.67	0.28	0.28	0.23	0.17
35	2,2,4,4-Tetramethyloctane	n.f.		-	-	-	-	-	-	-	-
36	D-Limonene	34	c	Fruity, lemon-like	0.50	0.60	0.31	0.14	0.15	0.11	0.09
37	2-Methyl-6-methylene-3,7-octadien-2-ol	n.f.		-	-	-	-	-	-	-	-
38	Pyrazine	180,000	c	Nutty, burnt	0.00	0.00	0.00	0.00	0.00	0.00	0.00
39	Benzyl alcohol	20,000	a	Sweet, floral, rose-like, caramel-like	0.00	0.00	0.00	0.00	0.00	0.00	0.00
40	*β*-Ocimene	0.02	a	Warm, floral, herbal, sweet	1485.87	2020.19	1225.20	578.85	635.82	480.95	455.01
41	(*Z*)-3-hexenyl crotonate	n.f.		Green, vegetable	-	-	-	-	-	-	-
42	*β*-Terpinene	85	c	Citrusy, woody, lemon-like	0.14	0.48	0.29	0.14	0.15	0.11	0.11
43	Formic acid, octyl ester	n.f.		Fruity, rose, orange, waxy, cucumber	-	-	-	-	-	-	-
44	3,5-Octadien-2-one	0.5	i	Creamy and fruity smell	26.59	42.60	29.28	13.35	22.64	10.44	3.63
45	Linalool oxide	190	a	Sweet, floral, creamy	0.05	0.11	0.06	0.03	0.03	0.02	0.02
46	3-Nonanone	17	h	Fresh, sweet Jasmine, spicy leaf, herbal, fruity	0.01	0.01	0.01	0.00	0.00	0.00	0.00
47	1,2-Dimethyl-4-vinylbenzene	n.f.		-	-	-	-	-	-	-	-
48	Linalool	0.22	a	Floral, sweet, grape-like	641.97	958.49	497.16	213.70	209.02	169.52	145.13
49	3,7-Dimethyl-1,5,7-octatriene-3-ol	n.f.		Moldy	-	-	-	-	-	-	-
50	2,3,4-Trimethyl-2-cyclopenten-1-one	n.f.		-	-	-	-	-	-	-	-
51	Phenylethyl alcohol	390	c	Floral, rose-like	0.13	0.15	0.14	0.04	0.04	0.03	0.04
52	(*E*, *E*)-2,6-Dimethyl-1,3,5,7-octatetraene	n.f.		-	-	-	-	-	-	-	-
53	(*E*, *Z*)-2,6-Dimethylocta-2,4,6-triene	n.f.		-	-	-	-	-	-	-	-
54	1,3,8-p-Menthatriene	n.f.		Turpentine, camphor, herbal, woody	-	-	-	-	-	-	-
55	1-Dodecanol	1000	h	Earthy, soapy, waxy, fatty, honey, coconut	0.00	0.00	0.00	0.00	0.00	0.00	0.00
56	5-Ethyldecane	n.f.		-	-	-	-	-	-	-	-
57	5-Ethyl-6-methyl-3(*E*)-hepten-2-one	n.f.		-	-	-	-	-	-	-	-
58	5-Methylundecane	n.f.		-	-	-	-	-	-	-	-
59	3-Methylundecane	n.f.		-	-	-	-	-	-	-	-
60	1-Nonanol	310	h	Fresh, clean, fatty, floral	0.02	0.01	0.01	0.00	0.00	0.00	0.00
61	*cis*-Linalool oxide (pyranoid)	190	a	Sweet, floral, creamy	0.03	0.05	0.04	0.01	0.01	0.01	0.01
62	(*E*)-3-Hexenyl butanoate	n.f.		-	-	-	-	-	-	-	-
63	1-Dodecene	1,280,000	h	-	0.00	0.00	0.00	0.00	0.00	0.00	0.00
64	Octanoic acid, ethyl ester	n.f.		-	-	-	-	-	-	-	-
65	Dodecane	10,000	c	Alkane-like	0.00	0.00	0.00	0.00	0.00	0.00	0.00
66	Decanal	5	g	Pungent, green, and floral odor	2.67	1.70	1.22	0.07	0.04	0.04	0.81
67	(*E*)-2-Hexenyl butanoate	n.f.		Green, fruity, orchid	-	-	-	-	-	-	-
68	*α*-Terpineol	330	c	Pleasant, floral	0.01	0.01	0.01	0.00	0.00	0.00	0.00
69	(*E*)-3-Dodecene	n.f.		-	-	-	-	-	-	-	-
70	Methyl salicylate	40	a	Minty, fresh, sweet	3.15	5.47	5.52	2.35	2.99	1.55	1.34
71	*β*-Cyclocitral	3	c	Citrus-like	23.07	40.40	41.91	17.42	22.34	11.17	9.58
72	Safranal	3	c	Woody, spicy, phenolic	0.04	0.05	0.04	0.02	0.02	0.03	0.07
73	Bis(2-ethylhexyl) carbonate	n.f.		-	-	-	-	-	-	-	-
74	7-Methyl-3-methylene-6-octen-1-ol	n.f.		-	-	-	-	-	-	-	-
75	2-Methyl-5-isopropenyl1-cyclopenten-1-carboxaldehyde	n.f.		-	-	-	-	-	-	-	-
76	(*Z*)-3-Hexenyl pentanoate	n.f.		Green	-	-	-	-	-	-	-
77	Quinoline	710	h	Earthy, musty, nutty	0.00	0.00	0.00	0.00	0.00	0.00	0.00
78	4,5-Dimethyl-2,6-octadiene	n.f.		-	-	-	-	-	-	-	-
79	*β*-Citral	53	c	Citrus, lemon-like	7.23	11.46	8.07	4.86	5.51	4.39	3.53
80	Caprolactam	59,700	h	Amine, spicy	0.00	0.00	0.00	0.00	0.00	0.00	0.00
81	Citral	30	h	Citrus, lemon-like	9.43	22.84	0.88	0.58	0.66	3.53	2.22
82	Ethyl salicylate	115	h	Sweet, curative, phenolic	0.00	0.01	0.03	0.00	0.01	0.00	0.00
83	3,7-Dimethyl-cis-2,6-octadienyl formate	n.f.		Green, rose, geranium, herbal, fruity	-	-	-	-	-	-	-
84	Indole	40	c	Floral, animal-like	0.27	0.03	0.01	0.00	0.00	0.00	0.00
85	(*E*)-Methyl 3,7-dimethylocta-2,6-dienoate	n.f.		Waxy, green, fruity, flower	-	-	-	-	-	-	-
86	Dimethyl salicylate	n.f.		Herbal, floral, fruity	-	-	-	-	-	-	-
87	Geranic acid	n.f.		Acidic, green, moldy, woody	-	-	-	-	-	-	-
88	*α*-Cubebene	14	j	Herb, wax	0.04	0.06	0.06	0.03	0.03	0.03	0.02
89	2-Butyl-2-octenal	20	h	Green, vegetable	0.03	0.06	0.03	0.00	0.00	0.00	0.00
90	(*Z*)-3-Hexenyl hexanoate	n.f.		Fruity, green, waxy, pear, winey, tropical grassy pineapple	-	-	-	-	-	-	-
91	(*Z*)-3-Hexenyl (*Z*)-3-hexenoate	781	c	Green, waxy, winey, grassy	0.00	0.01	0.01	0.00	0.00	0.00	0.01
92	Tetradecane	10,000	c	Alkane-like	0.00	0.00	0.00	0.00	0.00	0.00	0.00
93	(*Z*)-Jasmone	7	c	Jasmine-like, herbal, floral, woody	0.80	0.53	0.29	0.13	0.14	0.08	0.08
94	*α*-Ionone	76	a	Floral, violet-like, powdery, berry-like	0.02	0.03	0.02	0.01	0.01	0.00	0.00
95	Caryophyllene	64	c	Woody, green, spicy	0.01	0.02	0.01	0.00	0.01	0.00	0.00
96	Geranyl acetone	n.f.		Floral, green, magnolia, fruity	-	-	-	-	-	-	-
97	*β*-lonone	0.01	a	Violet-like, raspberry, floral	895.11	1759.80	1136.86	631.48	780.28	409.87	379.56
98	(*Z*)-Calamenene	n.f.		-	-	-	-	-	-	-	-
99	(R)-4,4,7a-Trimethyl-5,6,7,7a-tetrahydrobenzofuran-2(4H)-one (Dihydroactinidiolide)	n.f.	i	Floral, rose-like	-	-	-	-	-	-	-
100	*β*-Calacorene	n.f.		-	-	-	-	-	-	-	-
101	3-Methylpentadecane	n.f.		-	-	-	-	-	-	-	-
102	*α*-Nerolidol	10	i	Slight neroli-like, rose-like, and sweet	0.13	0.17	0.17	0.10	0.10	0.07	0.07
103	(*Z*)-3-Hexenyl benzoate	500	c	Fresh, green, leafy	0.00	0.00	0.00	0.00	0.00	0.00	0.00
104	Hexadecane	13,000,000	c	Alkane-like	0.00	0.00	0.00	0.00	0.00	0.00	0.00
105	Cadalene	n.f.		-	-	-	-	-	-	-	-
106	Caffeine	n.f.		Odorless	-	-	-	-	-	-	-

All odor thresholds were obtained from: g, [31]; a, [32]; b, [33]; c, [34]; d, [35]; e, [36]; f, [37]; i, [38]; j, [39]; k, [40]; h, [41]. “n.f.”: Data were not found in the literature. The odor descriptions of compounds were referred from http://www.thegoodscentscompany.com/search2.html (accessed on 15 November 2022).

## Data Availability

The original contributions presented in the study are included in the article/supplementary material, further inquiries can be directed to the corresponding authors.

## Supplementary Materials

The following supporting information can be downloaded at: https://www.mdpi.com/article/10.3390/foods13050728/s1.

## References

[1] Wen M., Han Z., Cui Y., Ho C.-T., Wan X., Zhang L. (2022). Identification of 4-O-p-coumaroylquinic acid as astringent compound of Keemun black tea by efficient integrated approaches of mass spectrometry, turbidity analysis and sensory evaluation. Food Chem..

[2] Fang X., Liu Y., Xiao J., Ma C., Huang Y. (2023). GC–MS and LC-MS/MS metabolomics revealed dynamic changes of volatile and non-volatile compounds during withering process of black tea. Food Chem..

[3] Wang Y., Liu Y., Cui Q., Li L., Ning J., Zhang Z. (2021). Monitoring the withering condition of leaves during black tea processing via the fusion of electronic eye (E-eye), colorimetric sensing array (CSA), and micro-near-infrared spectroscopy (NIRS). J. Food Eng..

[4] Chen Q., Zhu Y., Liu Y., Liu Y., Dong C., Lin Z., Teng J. (2022). Black tea aroma formation during the fermentation period. Food Chem..

[5] Xue J., Yin P., Zhang J., Wang W., Chen L., Su W., Guo G., Jiang H. (2020). Research progress on quality-related chemical components and processing technology of Congou black tea. Food Res. Dev..

[6] Ho C.-T., Zheng X., Li S. (2015). Tea aroma formation. Food Sci. Hum. Wellness.

[7] Yu J., Liu Y., Zhang S., Luo L., Zeng L. (2021). Effect of brewing conditions on phytochemicals and sensory profiles of black tea infusions: A primary study on the effects of geraniol and beta-ionone on taste perception of black tea infusions. Food Chem..

[8] Kiyomichi D., Franc C., Moulis P., Riquier L., Ballestra P., Marchand S., Tempère S., de Revel G. (2023). Investigation into mousy off-flavor in wine using gas chromatography-mass spectrometry with stir bar sorptive extraction. Food Chem..

[9] Zurowietz A., Lehr P.P., Kleb M., Merkt N., Gödde V., Bednarz H., Niehaus K., Zörb C. (2022). Training grapevines generates a metabolomic signature of wine. Food Chem..

[10] Biancolillo A., Aloia R., Rossi L., D’Archivio A.A. (2022). Organosulfur volatile profiles in Italian red garlic (*Allium sativum* L.) varieties investigated by HS-SPME/GC-MS and chemometrics. Food Control.

[11] Su D., He J.-J., Zhou Y.-Z., Li Y.-L., Zhou H.-J. (2022). Aroma effects of key volatile compounds in Keemun black tea at different grades: HS-SPME-GC-MS, sensory evaluation, and chemometrics. Food Chem..

[12] Wen R., Kong B., Yin X., Zhang H., Chen Q. (2022). Characterisation of flavour profile of beef jerky inoculated with different autochthonous lactic acid bacteria using electronic nose and gas chromatography–ion mobility spectrometry. Meat Sci..

[13] Chen J., Yang Y., Deng Y., Liu Z., Xie J., Shen S., Yuan H., Jiang Y. (2022). Aroma quality evaluation of Dianhong black tea infusions by the combination of rapid gas phase electronic nose and multivariate statistical analysis. LWT.

[14] Yang Y., Qian M.C., Deng Y., Yuan H., Jiang Y. (2022). Insight into aroma dynamic changes during the whole manufacturing process of chestnut-like aroma green tea by combining GC-E-Nose, GC-IMS, and GC × GC-TOFMS. Food Chem..

[15] Yao W., Cai Y., Liu D., Chen Y., Li J., Zhang M., Chen N., Zhang H. (2022). Analysis of flavor formation during production of Dezhou braised chicken using headspace-gas chromatography-ion mobility spec-trometry (HS-GC-IMS). Food Chem..

[16] Parastar H., Weller P. (2024). Towards greener volatilomics: Is GC-IMS the new Swiss army knife of gas phase analysis?. TrAC Trends Anal. Chem..

[17] Guo S., Zhao X., Ma Y., Wang Y., Wang D. (2022). Fingerprints and changes analysis of volatile compounds in fresh-cut yam during yellowing process by using HS-GC-IMS. Food Chem..

[18] Zhang J., Zhang W., Zhou L., Zhang R. (2021). Study on the influences of ultrasound on the flavor profile of unsmoked bacon and its underlying metabolic mechanism by using HS-GC-IMS. Ultrason. Sonochem..

[19] Song J., Shao Y., Yan Y., Li X., Peng J., Guo L. (2021). Characterization of volatile profiles of three colored quinoas based on GC-IMS and PCA. LWT Food Sci. Technol..

[20] Wang S., Chen H., Sun B. (2020). Recent progress in food flavor analysis using gas chromatography-ion mobility spectrometry (GC-IMS). Food Chem..

[21] Lin X., Lu M. (2012). Adaptability of Processing into Congu Black Tea by Oolong Tea Cultivars Jinguanyin etc. J. Tea Commun..

[22] Xie J., Wang L., Deng Y., Yuan H., Zhu J., Jiang Y., Yang Y. (2023). Characterization of the key odorants in floral aroma green tea based on GC-E-Nose, GC-IMS, GC-MS and aroma recombination and investigation of the dynamic changes and aroma formation during processing. Food Chem..

[23] Yang Y., Xie J., Wang Q., Deng Y., Zhu L., Zhu J., Yuan H., Jiang Y. (2024). Understanding the dynamic changes of volatile and non-volatile metabolites in black tea during processing by integrated volatolomics and UHPLC-HRMS analysis. Food Chem..

[24] Wang J., Ouyang W., Zhu X., Jiang Y., Yu Y., Chen M., Yuan H., Hua J. (2023). Effect of shaking on the improvement of aroma quality and transformation of volatile metabolites in black tea. Food Chem. X.

[25] Jia J., Zhang C., Yuan B., Chen Z., Chen J. (2021). Development and process parameter optimization with an integrated test bench for rolling and forming strips of oolong tea. J. Food Process Eng..

[26] Ozdemir F., Tontul I., Balci-Torun F., Topuz A. (2017). Effect of rolling methods and storage on volatile constituents of Turkish black tea. Flavour Fragr. J..

[27] Wu H., Huang W., Chen Z., Chen Z., Shi J., Kong Q., Sun S., Jiang X., Chen D., Yan S. (2019). GC–MS-based metabolomic study reveals dynamic changes of chemical compositions during black tea processing. Food Res. Int..

[28] Li M., Yang R., Zhang H., Wang S., Chen D., Lin S. (2019). Development of a flavor fingerprint by HS-GC–IMS with PCA for volatile compounds of Tricholoma matsutake Singer. Food Chem..

[29] Yang Y., Xie J., Chen J., Deng Y., Shen S., Hua J., Wang J., Zhu J., Yuan H., Jiang Y. (2022). Characterization of N,O-heterocycles in green tea during the drying process and unraveling the formation mechanism. Food Control.

[30] Yang Z., Baldermann S., Watanabe N. (2013). Recent studies of the volatile compounds in tea. Food Res. Int..

[31] Liao X.L., Yan J.N., Wang B., Meng Q., Zhang L.Y., Tong H.R. (2020). Identification of key odorants responsible for cooked corn-like aroma of green teas made by tea cultivar ‘Zhonghuang 1’. Food Res. Int..

[32] Guo X., Ho C.-T., Wan X., Zhu H., Liu Q., Wen Z. (2021). Changes of volatile compounds and odor profiles in Wuyi rock tea during processing. Food Chem..

[33] Ni H., Jiang Q., Lin Q., Ma Q., Wang L., Weng S., Huang G., Li L., Chen F. (2021). Enzymatic hydrolysis and auto-isomerization during β-glucosidase treatment improve the aroma of instant white tea infusion. Food Chem..

[34] Guo X., Ho C.-T., Schwab W., Wan X. (2021). Effect of the roasting degree on flavor quality of large-leaf yellow tea. Food Chem..

[35] Flaig M., Qi S., Wei G., Yang X., Schieberle P. (2020). Characterization of the Key Odorants in a High-Grade Chinese Green Tea Beverage (*Camellia sinensis*; Jingshan cha) by Means of the Sensomics Approach and Elucidation of Odorant Changes in Tea Leaves Caused by the Tea Manufacturing Process. J. Agric. Food Chem..

[36] Sánchez-Palomo E., Trujillo M., García Ruiz A., González Viñas M.A. (2017). Aroma profile of malbec red wines from La Mancha region: Chemical and sensory characterization. Food Res. Int..

[37] Zhang W., Cao J., Li Z., Li Q., Lai X., Sun L., Chen R., Wen S., Sun S., Lai Z. (2021). HS-SPME and GC/MS volatile component analysis of Yinghong No. 9 dark tea during the pile fermentation process. Food Chem..

[38] Zhu J., Chen F., Wang L., Niu Y., Yu D., Shu C., Chen H., Wang H., Xiao Z. (2015). Comparison of Aroma-Active Volatiles in Oolong Tea Infusions Using GC–Olfactometry, GC–FPD, and GC–MS. J. Agric. Food Chem..

[39] Yang C., Luo L., Zhang H., Yang X., Lv Y., Song H. (2010). Common aroma-active components of propolis from 23 regions of China. J. Sci. Food Agric..

[40] Uselmann V., Schieberle P. (2015). Decoding the Combinatorial Aroma Code of a Commercial Cognac by Application of the Sensomics Concept and First Insights into Differences from a German Brandy. J. Agric. Food Chem..

[41] Van Gemert L.J. (2011). ODOUR THRESHOLDS—Compilations of Odour Threshold Values in Air, Water and Other Media.

