# Supplementary material for: Uncovering the Dynamic Alterations of Volatile Components in Sweet and Floral Aroma Black Tea during Processing
Source: Foods. 2024 Feb 28;13(5):728. doi: 10.3390/foods13050728 (PMC10930892; doi:10.3390/foods13050728)
Supplement: Supplementary file 1 [file foods-13-00728-s001.zip › foods-2820047-supplementary.pdf]

Table S1 The information of chemicals used in this study.

Chemicals	Manufacturer information
Ethyl decanoate	TCI Chemical Industry Development Co., Ltd
High purity water	Hangzhou Wahaha Group Co., Ltd.
n-Alkane mixtures (C7-C40)	Shanghai Yuanye Biotechnology Co., Ltd.

Table S2 The identified volatile compounds in sweet and floral aroma black tea during the entire manufacturing process by using GC-IMS.

No.	Compounds	Formulas	MW	RI	Rt [sec]	Dt [a.u.]	Types	Peak intensities							Comment
								Fresh leaves	Withering	Shaking	Rolling	Fermentation	First drying	Final firing	
#3	Linalool	$C_{10}H_{18}O$	154.3	1102.1	497.755	1.21735	Alcohols	4197.53 ± 318.58d	6624.05 ± 211.01b	7584.27 ± 152.96a	6017.60 ± 90.58c	5979.39 ± 68.58c	3424.66 ± 59.03e	4269.92 ± 96.77d	
#32	2-Butoxyethanol	$C_6H_{14}O_2$	118.2	898.8	262.525	1.19921	Alcohols	82.27 ± 11.63c	104.16 ± 8.56b	128.79 ± 9.22a	62.82 ± 5.44d	97.06 ± 8.70b	37.96 ± 2.20e	41.10 ± 2.28e	
#36	(E)-2-Hexen-1-ol	$C_6H_{12}O$	100.2	848.3	233.41	1.51715	Alcohols	2460.34 ± 50.04c	2208.67 ± 44.94d	2533.77 ± 44.44bc	2586.43 ± 63.06b	2979.77 ± 57.78a	1929.30 ± 48.16e	1782.84 ± 50.61f	
#46	(E)-3-Hexen-1-ol	$C_6H_{12}O$	100.2	866.9	243.374	1.23957	Alcohols	542.42 ± 33.75d	567.47 ± 34.66d	612.00 ± 18.12c	766.70 ± 6.53b	804.38 ± 15.55b	1135.88 ± 23.00a	1092.52 ± 32.23a	
#68	Pentan-1-ol-M	$C_5H_{12}O$	88.1	762.7	190.332	1.25248	Alcohols	390.45 ± 5.60a	368.40 ± 7.50b	295.79 ± 6.74c	139.40 ± 0.10f	159.42 ± 4.50e	217.80 ± 5.47d	224.22 ± 7.92d	Monomer
#78	Pentan-1-ol-D	$C_5H_{12}O$	88.1	762.9	190.397	1.50823	Alcohols	620.69 ± 17.54c	808.90 ± 18.55a	651.11 ± 30.01b	285.62 ± 4.80d	266.76 ± 9.91d	112.65 ± 3.86f	161.01 ± 11.22e	Dimer
#79	3-Methylbutan-1-ol-D	$C_5H_{12}O$	88.1	727.3	175.966	1.48408	Alcohols	146.14 ± 2.20b	203.68 ± 3.72a	198.25 ± 6.58a	124.35 ± 2.23c	103.49 ± 3.93d	26.15 ± 1.28e	22.37 ± 0.27e	Dimer
#82	2-Methyl-1-propanol	$C_4H_{10}O$	74.1	624	142.881	1.36641	Alcohols	417.28 ± 9.68c	707.02 ± 22.53a	584.19 ± 8.70b	193.18 ± 0.85d	165.94 ± 3.31e	59.88 ± 7.50f	39.28 ± 2.11g	
#91	3-Methylbutan-1-ol-M	$C_5H_{12}O$	88.1	729.8	176.98	1.24282	Alcohols	38.10 ± 5.38bc	37.30 ± 2.05bc	37.84 ± 1.67bc	42.45 ± 1.61b	54.20 ± 1.95a	33.31 ± 0.78cd	32.05 ± 2.27d	Monomer

#92	2-Methylbutan-1-ol-M	$C_5H_{12}O$	88.1	732.4	178.047	1.22947	Alcohols	9.43 ± 3.01bc	14.41 ± 0.39a	13.72 ± 0.60a	10.66 ± 0.67b	9.79 ± 0.23b	8.85 ± 0.90bc	7.15 ± 1.01c	Monomer
#93	Pentan-2-ol	$C_5H_{12}O$	88.1	711.6	169.626	1.45245	Alcohols	50.08 ± 4.16c	39.33 ± 2.68c	83.04 ± 0.50b	318.04 ± 12.48a	303.48 ± 15.30a	42.70 ± 2.87c	24.57 ± 4.34d	
#96	tert-Butanol	$C_4H_{10}O$	74.1	508.9	111.816	1.14369	Alcohols	205.17 ± 14.14d	287.84 ± 4.32c	290.77 ± 7.36c	346.03 ± 20.33b	502.28 ± 3.61a	363.08 ± 19.92b	134.52 ± 6.70e	
#97	*n*-Hexanol	$C_6H_{14}O$	102.2	873.8	247.073	1.32819	Alcohols	306.55 ± 9.83b	394.72 ± 5.82a	233.06 ± 6.00d	234.77 ± 3.66d	251.96 ± 3.78c	242.72 ± 10.14cd	205.00 ± 6.66e	
#98	2-Methylbutan-1-ol-D	$C_5H_{12}O$	88.1	724.2	174.74	1.46541	Alcohols	110.32 ± 0.75b	139.30 ± 11.07a	139.68 ± 4.67a	115.55 ± 5.65b	85.93 ± 2.30c	21.28 ± 1.06d	22.55 ± 3.38d	Dimer
#12	(E, E)-2,4-Heptadienal	$C_7H_{10}O$	110.2	1011.1	366.97	1.19696	Aldehydes	242.65 ± 10.58e	369.57 ± 8.33c	503.59 ± 26.27b	463.64 ± 42.30b	696.06 ± 45.22a	497.47 ± 12.90b	291.73 ± 4.72d	
#20	Benzaldehyde-M	C_7H_6O	106.1	957.1	312.341	1.14868	Aldehydes	1624.48 ± 22.83b	1660.72 ± 7.69a	1445.84 ± 8.64c	713.06 ± 13.04ef	735.44 ± 22.34e	697.23 ± 2.37f	1208.33 ± 4.81d	Monomer
#21	Benzaldehyde-D	C_7H_6O	106.1	957.1	312.341	1.46512	Aldehydes	4647.47 ± 55.90a	4610.32 ± 16.57a	2960.66 ± 59.22b	343.06 ± 15.73d	339.22 ± 10.67d	231.70 ± 5.90e	831.77 ± 7.93c	Dimer
#25	Heptanal-M	$C_7H_{14}O$	114.2	899.4	263.11	1.3379	Aldehydes	334.58 ± 8.14g	429.66 ± 9.06c	353.94 ± 0.53f	390.10 ± 7.69e	407.33 ± 6.54d	636.53 ± 12.39a	524.25 ± 8.14b	Monomer
#26	Heptanal-D	$C_7H_{14}O$	114.2	898.8	262.531	1.69914	Aldehydes	333.20 ± 7.62e	678.68 ± 21.98b	528.88 ± 19.97d	539.89 ± 22.51d	571.95 ± 20.66cd	970.29 ± 43.51a	612.64 ± 24.27c	Dimer
#33	(E)-Hept-2-enal	$C_7H_{12}O$	112.2	951.1	307.204	1.25402	Aldehydes	59.98 ± 3.40c	56.69 ± 0.57c	73.66 ± 1.66b	62.28 ± 4.04c	93.81 ± 5.49a	68.17 ± 3.56b	28.89 ± 2.59d	Unidentified
#42	Octanal	$C_8H_{16}O$	128.2	1004.1	356.92	1.4119	Aldehydes	77.12 ± 6.58c	100.09 ± 5.82ab	107.24 ± 5.83a	42.31 ± 0.44d	44.50 ± 3.92d	103.55 ± 7.81ab	96.77 ± 4.05b	
#45	Nonanal	$C_9H_{18}O$	142.2	1107.6	505.747	1.4811	Aldehydes	260.91 ± 16.71c	217.58 ± 14.43d	240.52 ± 5.27cd	169.26 ± 13.46e	187.61 ± 9.40e	398.32 ± 21.96a	370.41 ± 17.11b	

#49	Hexanal-M	$C_6H_{12}O$	100.2	790	202.166	1.26454	Aldehydes	375.24 ± 4.56de	364.57 ± 7.35ef	357.82 ± 3.54f	404.74 ± 8.94c	379.42 ± 3.64d	654.44 ± 5.20a	505.86 ± 8.75b	Monomer
#50	Hexanal-D	$C_6H_{12}O$	100.2	791.6	203.03	1.56406	Aldehydes	558.22 ± 40.85e	524.33 ± 7.97e	691.35 ± 11.25d	1560.52 ± 25.34b	1602.18 ± 14.51ab	1649.07 ± 48.68a	1034.17 ± 13.45c	Dimer
#52	(E)-2-Pentenal-M	C_5H_8O	84.1	750	185.17	1.10704	Aldehydes	167.83 ± 4.40f	126.74 ± 2.40g	200.59 ± 6.52e	373.04 ± 4.63d	385.83 ± 0.95c	572.04 ± 9.32a	493.03 ± 13.92b	Monomer
#53	(E)-2-Pentenal-D	C_5H_8O	84.1	748.8	184.703	1.36125	Aldehydes	659.10 ± 41.11e	597.51 ± 22.76f	1193.92 ± 2.38c	1394.62 ± 40.34b	1624.23 ± 33.64a	787.80 ± 2.13d	689.54 ± 9.71e	Dimer
#57	Pentanal-M	$C_5H_{10}O$	86.1	695	162.869	1.18413	Aldehydes	119.99 ± 1.34b	107.49 ± 4.36c	70.67 ± 2.68e	84.38 ± 3.08d	81.81 ± 2.78d	194.45 ± 0.81a	120.06 ± 2.82b	Monomer
#58	Pentanal-D	$C_5H_{10}O$	86.1	693	162.078	1.4225	Aldehydes	304.78 ± 12.88c	331.11 ± 3.06b	285.87 ± 3.11d	240.04 ± 4.02e	279.09 ± 11.97d	353.63 ± 15.88a	144.13 ± 7.03f	Dimer
#59	2-Methylbutanal	$C_5H_{10}O$	86.1	660.9	152.809	1.39939	Aldehydes	1905.56 ± 17.68f	2362.14 ± 18.97d	2271.56 ± 19.60e	2454.12 ± 16.13c	2369.34 ± 8.06d	2975.94 ± 46.82b	3139.16 ± 43.45a	
#60	3-Methylbutanal	$C_5H_{10}O$	86.1	640.5	147.308	1.41143	Aldehydes	1289.39 ± 42.46f	1516.07 ± 5.94e	1502.55 ± 14.43e	1697.11 ± 9.25c	1581.10 ± 15.29d	1814.31 ± 33.62b	1944.14 ± 9.08a	
#64	Butanal	C_4H_8O	72.1	548.7	122.555	1.28401	Aldehydes	1124.16 ± 35.30e	1538.96 ± 38.81b	1745.00 ± 26.93a	1532.83 ± 20.39b	1304.15 ± 6.62d	1294.82 ± 43.71d	1405.48 ± 120.81c	
#76	2-Methyl-2-pentenal-D	$C_6H_{10}O$	98.1	823	219.874	1.49791	Aldehydes	405.34 ± 27.22c	608.65 ± 21.67a	534.71 ± 16.00b	65.08 ± 2.81e	98.58 ± 4.79d	34.42 ± 3.37f	53.69 ± 2.80ef	Dimer
#81	2-Methyl-2-pentenal-M	$C_6H_{10}O$	98.1	822.2	219.396	1.16162	Aldehydes	118.81 ± 5.42c	138.54 ± 2.37a	125.03 ± 4.64b	50.20 ± 3.39e	59.32 ± 1.21d	33.27 ± 1.78f	34.00 ± 1.61f	Monomer
#1	(Z)-3-Hexenyl butyrate	$C_{10}H_{18}O_2$	170.3	1240.4	696.712	1.43164	Esters	100.06 ± 5.19e	122.72 ± 5.08e	251.05 ± 20.48b	364.28 ± 10.29a	364.96 ± 28.79a	170.21 ± 2.46d	208.81 ± 15.50c	
#2	Methyl Salicylate	$C_8H_8O_3$	152.1	1230.1	681.86	1.20064	Esters	349.49 ± 51.70e	990.68 ± 54.19d	1501.37 ± 58.96c	1559.08 ± 94.92bc	1886.24 ± 53.57a	925.23 ± 121.12d	1673.94 ± 20.71b	

#14	Butanoic acid, 2-ethyl-3-methyl, ethyl ester-M	$C_9H_{18}O_2$	158.2	981	332.671	1.38724	Esters	1377.86 ± 34.04de	1361.29 ± 7.62e	1405.68 ± 13.21d	1818.18 ± 20.25c	1255.55 ± 45.35f	2324.57 ± 8.38a	2150.03 ± 11.66b	Monomer
#15	Butanoic acid, 2-ethyl-3-methyl, ethyl ester-D	$C_9H_{18}O_2$	158.2	982.3	333.802	1.82352	Esters	2385.39 ± 55.11d	3684.75 ± 33.84a	3345.28 ± 38.72b	1902.76 ± 99.91f	608.70 ± 41.12g	2227.20 ± 91.34e	3164.57 ± 107.40c	Dimer
#22	Ethyl 3-hydroxybutanoate	$C_6H_{12}O_3$	132.2	938.1	296.123	1.17298	Esters	317.60 ± 6.59d	336.74 ± 2.84c	315.70 ± 10.19d	590.82 ± 10.50b	767.55 ± 8.07a	140.09 ± 9.67e	83.41 ± 5.97f	
#23	Hexyl formate-M	$C_7H_{14}O_2$	130.2	921.5	281.934	1.28921	Esters	73.47 ± 2.00f	78.00 ± 1.90f	229.27 ± 6.93e	994.01 ± 10.54b	1025.64 ± 22.00a	555.54 ± 9.02c	520.70 ± 6.95d	Monomer
#24	Hexyl formate-D	$C_7H_{14}O_2$	130.2	920.1	280.775	1.6803	Esters	53.15 ± 5.17f	56.22 ± 2.42f	202.21 ± 2.96e	2435.46 ± 31.66a	2132.44 ± 26.51b	263.14 ± 4.02d	292.17 ± 6.71c	Dimer
#44	Ethyl hexanoate	$C_8H_{16}O_2$	144.2	1001.7	353.466	1.3425	Esters	35.90 ± 3.07c	22.92 ± 2.22e	26.57 ± 1.05de	80.44 ± 4.39a	58.40 ± 3.47b	27.76 ± 2.19de	29.39 ± 1.35d	
#55	n-Propyl acetate-D	$C_5H_{10}O_2$	102.1	707	167.771	1.47689	Esters	130.17 ± 5.23d	84.81 ± 1.04e	144.72 ± 7.00cd	1042.50 ± 23.96a	1047.13 ± 11.37a	327.49 ± 24.95b	159.36 ± 15.73c	Dimer
#56	n-Propyl acetate-M	$C_5H_{10}O_2$	102.1	706.7	167.613	1.16446	Esters	101.31 ± 4.58d	101.55 ± 4.20d	86.60 ± 2.18e	139.08 ± 2.03c	149.82 ± 3.98b	170.10 ± 5.97a	98.83 ± 3.73d	Monomer
#61	Ethyl Acetate	$C_4H_8O_2$	88.1	608.4	138.664	1.33719	Esters	983.29 ± 5.26d	781.11 ± 10.65e	1239.55 ± 12.18c	4427.55 ± 46.08a	4493.10 ± 60.38a	2900.81 ± 125.47b	661.58 ± 26.40f	
#66	Methyl acetate	$C_3H_6O_2$	74.1	537	119.411	1.19298	Esters	2076.91 ± 41.91e	3281.93 ± 51.25b	3462.17 ± 82.82a	3156.05 ± 105.14b	2778.39 ± 20.20c	2579.62 ± 142.72d	2794.58 ± 150.64c	
#77	Methyl valerate	$C_6H_{12}O_2$	116.2	825.4	221.138	1.564	Esters	184.58 ± 3.42c	265.38 ± 11.24a	229.94 ± 8.01b	85.13 ± 4.75e	112.33 ± 7.51d	26.46 ± 2.25f	22.74 ± 0.97f	
#84	Methyl butyrate-D	$C_5H_{10}O_2$	102.1	715.5	171.206	1.42264	Esters	223.70 ± 12.91d	223.49 ± 8.60d	346.64 ± 2.97c	724.64 ± 12.16a	490.30 ± 8.65b	137.54 ± 5.71e	124.28 ± 7.93e	Dimer

#87	Methyl 3-methylbutanoate	$C_6H_{12}O_2$	116.2	768.2	192.552	1.53234	Esters	47.29 ± 2.46e	72.17 ± 3.35c	81.77 ± 5.22b	131.89 ± 0.95a	60.74 ± 1.90d	9.83 ± 1.35f	11.66 ± 2.73f	
#88	Methyl butyrate-M	$C_5H_{10}O_2$	102.1	717.2	171.868	1.15016	Esters	56.82 ± 3.08g	64.72 ± 2.38f	102.63 ± 1.25c	247.44 ± 3.64a	203.00 ± 2.20b	93.22 ± 4.79d	75.80 ± 3.47e	Monomer
#89	Butyl acetate-M	$C_6H_{12}O_2$	116.2	803.5	209.421	1.23849	Esters	58.56 ± 1.11d	50.87 ± 1.15e	58.60 ± 0.93d	95.98 ± 1.46a	70.73 ± 4.66c	78.65 ± 2.04b	75.99 ± 1.71b	Monomer
#100	Butyl acetate-D	$C_6H_{12}O_2$	116.2	802.5	208.857	1.61975	Esters	9.75 ± 0.12d	10.27 ± 0.69d	11.89 ± 0.61d	76.35 ± 2.10a	75.03 ± 2.69a	24.36 ± 0.50b	14.98 ± 0.59c	Dimer
#4	*trans*-Linalool oxide-M	$C_{10}H_{18}O_2$	170.3	1084	471.749	1.2622	Heterocycles	758.03 ± 44.00e	1825.64 ± 37.04c	2009.57 ± 68.25a	1874.56 ± 59.01bc	1956.22 ± 22.98ab	1316.65 ± 36.34d	1900.77 ± 54.92bc	Monomer
#5	*trans*-Linalool oxide-D	$C_{10}H_{18}O_2$	170.3	1083.7	471.372	1.81672	Heterocycles	161.61 ± 27.97d	702.53 ± 40.82b	961.05 ± 46.39a	661.56 ± 36.27b	680.84 ± 21.23b	297.56 ± 22.04c	672.22 ± 46.71b	Dimer
#6	2,3-Dimethyl-5-ethylpyrazine	$C_8H_{12}N_2$	136.2	1069.3	450.642	1.23366	Heterocycles	738.73 ± 33.66a	678.81 ± 25.98b	743.55 ± 16.90a	430.63 ± 15.39d	584.38 ± 31.71c	253.16 ± 29.35e	212.85 ± 10.48e	
#7	*cis*-Linalool Oxide-M	$C_{10}H_{18}O_2$	170.3	1065.4	444.989	1.25948	Heterocycles	300.43 ± 9.25d	810.65 ± 17.82ab	815.78 ± 26.51ab	793.01 ± 36.69ab	820.78 ± 10.40a	504.27 ± 23.63c	775.16 ± 18.81b	Monomer
#8	*cis*-Linalool Oxide-D	$C_{10}H_{18}O_2$	170.3	1065.9	445.742	1.81129	Heterocycles	56.73 ± 8.38f	207.18 ± 22.20b	230.27 ± 8.85a	132.15 ± 8.21d	153.79 ± 15.82c	56.70 ± 4.56f	92.27 ± 4.28e	Dimer
#17	2-Pentyl furan	$C_9H_{14}O$	138.2	993.1	343.037	1.25129	Heterocycles	218.00 ± 5.89d	199.92 ± 4.88e	258.79 ± 4.33b	240.07 ± 5.53c	357.34 ± 11.77a	135.28 ± 4.67f	193.31 ± 3.43e	
#29	2-Acetylfuran	$C_6H_6O_2$	110.1	907.9	270.326	1.11836	Heterocycles	63.26 ± 3.84d	53.14 ± 2.94e	76.90 ± 3.80c	97.47 ± 7.49b	106.64 ± 2.30a	63.65 ± 1.18d	93.89 ± 4.24b	
#47	Furfural-M	$C_5H_4O_2$	96.1	825.8	221.326	1.08524	Heterocycles	79.21 ± 8.50b	53.94 ± 3.63c	68.96 ± 4.18b	50.83 ± 1.90c	56.60 ± 6.89c	71.28 ± 9.23b	489.96 ± 7.22a	Monomer
#48	Furfural-D	$C_5H_4O_2$	96.1	824.7	220.75	1.33466	Heterocycles	87.51 ± 8.64b	61.26 ± 3.44c	84.86 ± 4.07b	24.09 ± 3.65d	21.81 ± 1.82d	23.31 ± 1.38d	227.41 ± 3.07a	Dimer

#13	Methyl-5-hepten-2-one	$C_8H_{14}O$	126.2	990.2	340.586	1.17521	Ketones	4850.83 ± 184.15a	4906.21 ± 41.21a	3800.81 ± 71.72b	2077.25 ± 53.39d	2425.70 ± 42.15c	667.94 ± 27.70f	836.22 ± 27.87e	
#31	2-Heptanone-M	$C_7H_{14}O$	114.2	890.7	256.143	1.26224	Ketones	136.77 ± 4.75e	186.67 ± 1.85bc	154.40 ± 3.70d	156.16 ± 4.05d	182.35 ± 4.44c	200.17 ± 4.86a	190.94 ± 2.13b	Monomer
#35	2-Heptanone-D	$C_7H_{14}O$	114.2	887.4	254.37	1.62948	Ketones	274.99 ± 12.97c	409.58 ± 7.32a	358.34 ± 10.02b	105.49 ± 5.78e	206.92 ± 1.47d	36.60 ± 3.94g	75.86 ± 5.85f	Dimer
#39	Dihydro-2(3h)-furanone	$C_4H_6O_2$	86.1	919.2	279.956	1.07948	Ketones	226.80 ± 27.33b	183.03 ± 5.67c	148.99 ± 5.12d	78.10 ± 5.98e	79.47 ± 7.04e	155.05 ± 10.81d	327.73 ± 1.80a	
#51	Mesityl oxide	$C_6H_{10}O$	98.1	790	202.166	1.44485	Ketones	3501.78 ± 32.03a	3520.86 ± 63.25a	2572.35 ± 46.44b	574.98 ± 6.25c	601.05 ± 6.19c	467.39 ± 3.43d	610.79 ± 13.40c	
#63	2-Butanone	C_4H_8O	72.1	583.6	131.984	1.24689	Ketones	3792.82 ± 29.72a	3812.36 ± 126.06a	3417.91 ± 38.76b	2691.36 ± 42.46c	2625.41 ± 14.11c	487.81 ± 23.00e	1900.21 ± 62.67d	
#71	3-Hydroxybutan-2-one	$C_4H_8O_2$	88.1	711.8	169.704	1.05584	Ketones	102.06 ± 6.05c	104.29 ± 2.36c	101.43 ± 4.58c	42.49 ± 0.51e	53.84 ± 4.65d	163.49 ± 1.91b	233.85 ± 3.18a	
#73	(E)-3-Penten-2-one	C_5H_8O	84.1	737	179.92	1.09113	Ketones	47.88 ± 2.32b	29.23 ± 0.39e	33.57 ± 0.78d	40.88 ± 0.25c	41.12 ± 2.24c	29.65 ± 0.71e	54.01 ± 1.30a	
#83	3-Pentanone	$C_5H_{10}O$	86.1	694.6	162.724	1.35332	Ketones	155.50 ± 5.27d	157.30 ± 4.08d	265.18 ± 3.34a	216.20 ± 3.25b	164.58 ± 4.48c	59.64 ± 1.98f	70.18 ± 1.30e	
#86	2-Hexanone	$C_6H_{12}O$	100.2	791.2	202.794	1.5026	Ketones	221.88 ± 15.77bc	235.16 ± 7.97ab	238.55 ± a	211.82 ± 4.25c	175.61 ± 7.22d	97.43 ± 3.72e	181.93 ± 9.11d	
#94	Acetone	C_3H_6O	58.1	488.6	106.359	1.11507	Ketones	10415.09 ± 113.11a	9632.76 ± 43.85b	8936.56 ± 20.67c	8070.92 ± 110.72d	7773.82 ± 33.39e	4016.98 ± 55.74g	5033.12 ± 148.60f	
#95	Cyclopentanone	C_5H_8O	84.1	785	199.494	1.33634	Ketones	137.55 ± 6.99b	123.94 ± 3.69c	116.32 ± 0.88c	101.32 ± 2.82d	101.24 ± 2.64d	175.35 ± 1.53a	123.95 ± 7.03c	
#99	2-Pentanone	$C_5H_{10}O$	86.1	689.3	160.591	1.37061	Ketones	91.89 ± 2.77a	75.08 ± 6.97c	95.80 ± 0.64a	79.23 ± 0.65bc	83.79 ± 2.48b	47.60 ± 1.13d	17.14 ± 0.42e	

#80	Ethylsulfide	$C_4H_{10}S$	90.2	700.6	165.14	1.04444	Sulfides	231.00 ± 10.86a	153.32 ± 3.50c	130.14 ± 3.14de	191.50 ± 1.39b	232.92 ± 11.15a	119.99 ± 5.97e	137.28 ± 3.44d	
#9	γ-Terpinene	$C_{10}H_{16}$	136.2	1051.8	425.39	1.22278	Terpenes	353.01 ± 7.87b	225.88 ± 8.28d	235.10 ± 12.31d	332.22 ± 2.38c	405.93 ± 17.93a	99.37 ± 5.61e	109.56 ± 3.89e	
#19	α-Phellandrene	$C_{10}H_{16}$	136.2	1001.5	353.049	1.22014	Terpenes	29.67 ± 0.37e	36.61 ± 1.72d	46.45 ± 4.28c	72.89 ± 4.41b	89.94 ± 4.00a	42.13 ± 2.71cd	45.04 ± 4.16c	
#27	α-Fenchene	$C_{10}H_{16}$	136.2	938.8	296.703	1.21853	Terpenes	59.41 ± 1.96d	70.89 ± 4.66c	60.06 ± 2.10d	124.36 ± 4.81b	176.55 ± 2.79a	49.30 ± 2.61e	35.89 ± 2.62f	
#10	1	/	/	1037.3	404.66	1.45519		314.09 ± 14.77a	244.12 ± 8.49c	229.29 ± 11.62c	280.79 ± 3.34b	300.53 ± 8.61a	183.80 ± 17.38d	167.19 ± 5.87d	Unidentified
#11	2	/	/	1026.9	389.584	1.24453		465.38 ± 11.92c	318.84 ± 4.35f	373.39 ± 2.17e	645.57 ± 12.63b	701.92 ± 14.75a	271.29 ± 4.71g	432.90 ± 16.84d	Unidentified
#16	3	/	/	986	336.956	1.76743		589.83 ± 5.59b	686.70 ± 1.13a	598.33 ± 8.05b	282.72 ± 6.15c	205.20 ± 15.82d	114.18 ± 7.83f	179.94 ± 3.81e	Unidentified
#18	4	/	/	999.1	349.637	1.20362		45.83 ± 3.21d	52.23 ± 0.93d	67.84 ± 8.37c	92.68 ± 11.12b	155.93 ± 3.96a	92.73 ± 6.27b	22.92 ± 1.80e	Unidentified
#28	5	/	/	927.8	287.347	1.19921		33.96 ± 3.38f	53.18 ± 1.01e	71.96 ± 2.65d	186.02 ± 2.74a	172.67 ± 3.43b	65.95 ± 3.93d	108.72 ± 5.65c	Unidentified
#30	6	/	/	895	259.334	1.14439		60.29 ± 1.51e	77.09 ± 2.34d	92.33 ± 3.45c	163.04 ± 8.29b	215.18 ± 3.93a	63.01 ± 3.00e	52.05 ± 2.63f	Unidentified
#34	7	/	/	899.2	262.88	1.60755		323.13 ± 13.35e	366.88 ± 2.42d	426.19 ± 9.05ab	433.80 ± 7.60ab	387.35 ± 16.34c	442.11 ± 2.67a	418.06 ± 3.21b	
#37	8	/	/	1041.7	410.973	1.20956		129.16 ± 30.02a	36.85 ± 6.58b	31.03 ± 5.18b	31.29 ± 1.41b	41.24 ± 2.75b	23.29 ± 0.97b	43.27 ± 6.28b	Unidentified
#38	9	/	/	914.4	275.848	1.41123		130.10 ± 19.63a	108.88 ± 4.90b	115.33 ± 2.68ab	41.00 ± 5.64c	39.01 ± 3.12c	51.29 ± 4.41c	103.58 ± 1.32b	Unidentified

#40	10	/	/	1007.5	361.799	1.82086	616.77 ± 58.86a	168.78 ± 12.37c	239.46 ± 18.12b	180.46 ± 7.76c	99.24 ± 1.58d	68.37 ± 11.82d	76.60 ± 10.89d	Unidentified
#41	11	/	/	1009.5	364.587	1.32981	1156.77 ± 12.26a	728.72 ± 2.16c	766.24 ± 32.58b	713.16 ± 8.36c	624.16 ± 26.96d	284.15 ± 23.29e	307.28 ± 9.58e	Unidentified
#43	12	/	/	920.8	281.311	1.59935	164.50 ± 3.37f	133.61 ± 2.25g	379.38 ± 2.47c	593.62 ± 10.19a	541.37 ± 4.26b	205.78 ± 15.79e	226.99 ± 6.70d	Unidentified
#54	13	/	/	732.5	178.089	1.33282	1051.63 ± 56.16a	642.44 ± 11.53c	716.12 ± 21.04b	319.85 ± 12.98e	434.40 ± 10.60d	106.52 ± 2.67g	196.63 ± 9.03f	Unidentified
#62	14	/	/	596	135.324	1.28903	1427.52 ± 8.44d	1025.42 ± 15.62e	1467.82 ± 4.50c	1889.59 ± 26.99a	1851.42 ± 4.07b	700.14 ± 13.98g	966.96 ± 30.87f	Unidentified
#65	15	/	/	528.3	117.054	1.23786	137.48 ± 1.33e	89.81 ± 1.85f	156.96 ± 2.74d	288.81 ± 3.42a	247.15 ± 0.62b	132.75 ± 6.45e	176.50 ± 15.75c	Unidentified
#67	16	/	/	740.4	181.295	1.42896	366.31 ± 9.00d	544.29 ± 2.68a	485.77 ± 7.42b	313.04 ± 8.74e	397.08 ± 5.92c	101.91 ± 6.50g	177.26 ± 6.85f	Unidentified
#69	17	/	/	739.4	180.902	1.15466	33.29 ± 2.67d	33.70 ± 1.78d	35.61 ± 2.05d	68.12 ± 2.96c	80.78 ± 3.09b	70.45 ± 5.57c	93.34 ± 1.58a	Unidentified
#70	18	/	/	808.2	211.942	1.1587	179.34 ± 5.14b	98.98 ± 5.12e	124.19 ± 1.20d	195.68 ± 2.38a	136.50 ± 2.91c	59.15 ± 2.78g	65.04 ± 1.51f	Unidentified
#72	19	/	/	734.1	178.741	1.07197	33.72 ± 2.87c	9.46 ± 1.45e	12.53 ± 1.24e	25.48 ± 2.56d	35.65 ± 0.94c	58.41 ± 1.31a	50.06 ± 2.16b	Unidentified
#74	20	/	/	805.1	210.264	1.04222	355.04 ± 18.51a	167.18 ± 3.92d	139.91 ± 2.05e	248.21 ± 4.30c	264.11 ± 5.60b	103.21 ± 5.21f	149.85 ± 5.93e	Unidentified
#75	21	/	/	810.3	213.051	1.48935	458.64 ± 11.23a	210.09 ± 10.62e	300.97 ± 2.61c	348.38 ± 10.58b	262.37 ± 9.19d	51.13 ± 2.51f	49.00 ± 2.16f	Unidentified
#85	22	/	/	767.4	192.24	1.47719	113.23 ± 4.55f	170.85 ± 3.18d	238.31 ± 4.23c	382.54 ± 13.92a	355.51 ± 7.00b	148.55 ± 4.50e	166.38 ± 11.87d	Unidentified

#90	23	/	/	827.4	222.218	1.26542	180.96 ± 2.51b	202.27 ± 6.06a	169.58 ± 0.74c	143.22 ± 4.28d	175.04 ± 4.46bc	88.37 ± 3.68f	113.99 ± 4.27e	Unidentified

Data were expressed as mean ± SD (n = 3).

MW represents molecular mass; RI represents relative retention index; Rt represents retention time; Dt represents relative migration time; The suffixe-M represents monomer of volatile components while the suffixe-D represents dimer. Values in a row followed by different letters are significantly different ($p < 0.05$).

Table S3 Characterization and contents of the volatile compounds of sweet and floral aroma black tea during the entire manufacturing process based on GC-MS.

No.	Compounds	Rt[a]	RI[b]	RI[c]	ID	Compound types	Contents (μg/L)							VIP	Significance
							Fresh leaves	Withering	Shaking	Rolling	Fermentation	First drying	Final firing		
7	2-Methylbutanol	10.73	711	718	MS, RI	Alcohols	0.11 ± 0.02b	0.32 ± 0.06a	0.12 ± 0.03b	0.02 ± 0.00c	0.03 ± 0.00c	0.02 ± 0.00c	0.01 ± 0.00c	0.96	0
10	(Z)-2-Pentenol	11.85	747	746	MS, RI	Alcohols	0.73 ± 0.08e	2.41 ± 0.42a	2.20 ± 0.66ab	1.41 ± 0.08cd	1.93 ± 0.32abc	1.66 ± 0.30bcd	1.14 ± 0.10de	1.16	0
14	(E)-3-Hexen-1-ol	14.9	844	855	MS, RI	Alcohols	43.06 ± 4.70a	31.88 ± 4.15b	26.14 ± 8.15b	12.79 ± 1.81c	12.10 ± 1.72c	8.13 ± 1.85c	6.24 ± 0.42c	0.98	0
15	1-Hexanol	15.28	856	858	MS, RI	Alcohols	6.85 ± 0.79a	5.11 ± 0.23b	3.99 ± 1.84b	1.91 ± 0.18c	1.82 ± 0.26c	1.31 ± 0.28c	0.93 ± 0.14c	0.95	0
19	2-Heptanol	16.43	893	888	MS, RI	Alcohols	1.47 ± 0.79b	4.88 ± 4.13a	0.44 ± 0.18b	0.34 ± 0.05b	0.45 ± 0.05b	0.37 ± 0.08b	0.31 ± 0.02b	0.98	0.029
23	1-Heptanol	19.11	975	970	MS, RI	Alcohols	4.21 ± 0.58a	2.84 ± 0.08b	0.86 ± 0.13c	0.35 ± 0.04d	0.34 ± 0.06d	0.28 ± 0.07d	0.24 ± 0.04d	0.96	0
24	1-Octen-3-ol	19.49	986	981	MS, RI	Alcohols	7.36 ± 1.50b	12.51 ± 4.19a	4.60 ± 1.07bc	1.93 ± 0.22c	2.31 ± 0.46c	1.96 ± 0.62c	1.28 ± 0.08c	0.90	0
33	2-Ethyl-1-hexanol	21.36	1031	1039	MS, RI	Alcohols	1.09 ± 0.06b	1.78 ± 0.69a	0.74 ± 0.14bc	0.34 ± 0.03c	0.34 ± 0.06c	0.34 ± 0.11c	0.22 ± 0.03c	0.88	0
37	2-Methyl-6-methylene-3,7-octadien-2-ol	21.57	1037	/	MS, RI	Alcohols	7.15 ± 1.30a	8.04 ± 2.27a	4.00 ± 0.85b	1.09 ± 0.05c	1.63 ± 0.41c	0.91 ± 0.32c	1.17 ± 0.25c	0.85	0

39	Benzyl alcohol	21.79	1044	1051	MS, RI	Alcohols	46.34 ± 14.84ab	48.67 ± 13.83a	33.61 ± 4.60b	6.81 ± 0.27c	7.94 ± 1.89c	5.13 ± 1.55c	6.32 ± 1.21c	0.86	0
48	Linalool	24.17	1111	1112	MS, RI	Alcohols	141.23 ± 12.82b	210.87 ± 53.68a	109.38 ± 23.19b	47.01 ± 5.73c	45.98 ± 7.98c	37.29 ± 12.64c	31.93 ± 2.08c	0.85	0
49	3,7-Dimethyl-1,5,7-octatriene-3-ol (Hotrienol)	24.17	1111	1106	MS, RI	Alcohols	163.46 ± 15.09b	245.50 ± 62.60a	91.32 ± 77.57bc	39.17 ± 32.13cd	35.81 ± 28.66cd	2.54 ± 0.94d	3.85 ± 1.03d	0.84	0
51	Phenylethyl alcohol	24.7	1126	1127	MS, RI	Alcohols	52.58 ± 6.42a	58.93 ± 16.62a	53.43 ± 9.01a	14.94 ± 0.62b	16.36 ± 4.14b	11.96 ± 3.69b	13.84 ± 2.73b	0.89	0
55	1-Dodecanol	25.17	1140	1470	MS, RI	Alcohols	1.16 ± 0.16b	1.90 ± 0.27a	0.69 ± 0.20c	0.09 ± 0.01d	0.14 ± 0.04d	0.06 ± 0.02d	0.05 ± 0.01d	0.88	0
60	1-Nonanol	26.36	1173	1176	MS, RI	Alcohols	6.72 ± 0.67a	4.60 ± 0.94b	1.94 ± 0.20c	0.64 ± 0.08d	0.61 ± 0.12d	0.49 ± 0.25d	0.37 ± 0.06d	0.95	0
68	*α*-Terpineol	27.36	1201	1202	MS, RI	Alcohols	2.01 ± 0.25b	2.93 ± 0.45a	1.91 ± 0.34b	0.88 ± 0.05c	0.94 ± 0.19c	0.75 ± 0.24c	0.66 ± 0.05c	0.86	0
74	7-Methyl-3-methylene-6-octen-1-ol	28.02	1223	1221	MS, RI	Alcohols	3.24 ± 0.29b	4.59 ± 0.29a	2.69 ± 0.50c	1.26 ± 0.16d	1.42 ± 0.29d	1.14 ± 0.34d	0.92 ± 0.09d	0.86	0
102	*α*-Nerolidol	37.66	1572	1566	MS, RI	Alcohols	1.31 ± 0.15ab	1.67 ± 0.59a	1.71 ± 0.41a	1.03 ± 0.09bc	1.04 ± 0.26bc	0.68 ± 0.28c	0.69 ± 0.04c	0.96	0.005
2	2-Methylpropanal	6.23	567	558	MS, RI	Aldehydes	0.08 ± 0.02d	0.76 ± 0.31b	1.23 ± 0.25a	0.44 ± 0.05c	0.64 ± 0.11bc	0.90 ± 0.15b	0.72 ± 0.04bc	1.21	0
4	3-Methylbutanal	8.18	630	634	MS, RI	Aldehydes	0.12 ± 0.04d	0.99 ± 0.37c	1.86 ± 0.27a	1.08 ± 0.11bc	1.79 ± 0.37a	1.84 ± 0.29a	1.49 ± 0.13ab	1.04	0
5	2-Methylbutanal	8.45	638	645	MS, RI	Aldehydes	0.25 ± 0.08e	3.43 ± 1.58bcd	5.93 ± 0.96a	2.06 ± 0.14d	3.06 ± 0.58cd	4.81 ± 0.86ab	4.34 ± 0.08bc	1.28	0

11	Hexanal	12.86	779	782	MS, RI	Aldehydes	2.49 ± 0.49c	7.96 ± 3.89a	3.63 ± 0.29bc	4.08 ± 0.55bc	7.94 ± 1.92a	6.82 ± 1.86ab	2.30 ± 0.23c	1.28	0.004
12	2-Ethyl-2-butenal	14.06	817	808	MS, RI	Aldehydes	0.37 ± 0.08b	1.06 ± 0.24b	0.25 ± 0.11b	1.81 ± 2.71b	6.06 ± 1.08a	0.29 ± 0.08b	0.41 ± 0.59b	1.43	0
13	(E)-2-Hexenal	14.9	844	850	MS, RI	Aldehydes	16.43 ± 1.71b	4.39 ± 2.06d	8.02 ± 0.94c	16.11 ± 0.11b	19.60 ± 3.16a	6.94 ± 1.30cd	3.90 ± 0.41d	1.76	0
21	Heptanal	16.52	899	903	MS, RI	Aldehydes	0.70 ± 0.13cd	1.87 ± 0.69a	0.76 ± 0.11bcd	1.04 ± 0.13bcd	1.35 ± 0.30ab	1.17 ± 0.42bc	0.46 ± 0.06d	1.29	0.003
22	Benzaldehyde	19	972	966	MS, RI	Aldehydes	70.81 ± 6.59a	71.39 ± 7.70a	26.65 ± 5.42b	6.83 ± 0.37c	10.36 ± 2.42c	6.52 ± 1.45c	6.32 ± 0.46c	0.88	0
31	Octanal	20.55	1008	1009	MS, RI	Aldehydes	0.55 ± 0.09a	0.64 ± 0.14a	0.25 ± 0.03b	0.18 ± 0.04b	0.20 ± 0.05b	0.23 ± 0.07b	0.14 ± 0.05b	0.91	0
66	Decanal	27.3	1199	1202	MS, RI	Aldehydes	13.37 ± 0.26a	8.49 ± 7.08ab	6.08 ± 2.08b	0.33 ± 0.01c	0.20 ± 0.06c	0.21 ± 0.04c	4.07 ± 0.64bc	1.00	0
71	*β*-Cyclocitral	27.55	1207	1210	MS, RI	Aldehydes	69.21 ± 3.21b	121.19 ± 3.08a	125.74 ± 26.97a	52.26 ± 4.25bc	67.02 ± 13.77b	33.52 ± 13.43cd	28.75 ± 3.19d	1.01	0
72	Safranal	27.69	1211	1201	MS, RI	Aldehydes	0.11 ± 0.01c	0.15 ± 0.01b	0.11 ± 0.02c	0.05 ± 0.00e	0.06 ± 0.01de	0.08 ± 0.02cd	0.21 ± 0.02a	1.68	0
75	2-Methyl-5-isopropenyl1-cyclopenten-1-carboxaldehyde	28.17	1228	1271	MS, RI	Aldehydes	0.51 ± 0.03b	1.03 ± 0.20a	0.91 ± 0.17a	0.37 ± 0.03bc	0.36 ± 0.06bc	0.20 ± 0.05c	0.18 ± 0.04c	0.94	0
79	*β*-Citral	29.3	1266	1255	MS, RI	Aldehydes	383.03 ± 97.69bc	607.54 ± 162.77a	427.69 ± 77.19b	257.35 ± 25.75cd	291.93 ± 55.99bcd	232.73 ± 68.83cd	186.95 ± 10.31d	0.89	0

81	Citral	29.73	1280	1276	MS, RI	Aldehydes	282.82 ± 243.33b	685.14 ± 81.48a	26.26 ± 4.71c	17.29 ± 1.31c	19.82 ± 4.29c	106.03 ± 165.72bc	66.47 ± 97.35bc	1.08	0
89	2-Butyl-2-octenal	32.69	1379	1378	MS, RI	Aldehydes	0.61 ± 0.09b	1.19 ± 0.35a	0.70 ± 0.15b	0.04 ± 0.00c	0.05 ± 0.01c	0.02 ± 0.01c	0.04 ± 0.02c	0.88	0
29	2,2,4,6,6-Pentamethylheptane	20.05	1002	995	MS, RI	Alkanes	17.91 ± 2.63a	11.98 ± 7.42b	6.40 ± 2.18c	0.30 ± 0.03d	0.42 ± 0.07d	0.80 ± 0.59d	0.09 ± 0.02d	0.92	0
30	Decane	20.26	1000	1000	MS, RI	Alkanes	3.94 ± 0.28a	3.19 ± 1.93a	1.72 ± 0.66b	0.14 ± 0.02c	0.14 ± 0.04c	0.19 ± 0.13c	0.12 ± 0.04c	0.88	0
35	2,2,4,4-Tetramethyloctane	21.48	1035	/	MS, RI	Alkanes	4.54 ± 0.68a	2.89 ± 1.83b	1.79 ± 0.55b	0.06 ± 0.01c	0.08 ± 0.02c	0.19 ± 0.15c	0.15 ± 0.13c	0.94	0
56	5-Ethyldecane	25.46	1148	1146	MS, RI	Alkanes	1.67 ± 0.31a	0.97 ± 0.72b	0.59 ± 0.21bc	0.02 ± 0.01c	0.02 ± 0.01c	0.05 ± 0.03c	0.04 ± 0.00c	0.95	0
58	5-Methylundecane	26.1	1166	1156	MS, RI	Alkanes	0.62 ± 0.18a	0.69 ± 0.45a	0.41 ± 0.12ab	0.11 ± 0.00b	0.13 ± 0.03b	0.09 ± 0.04b	0.06 ± 0.00b	0.82	0.003
59	3-Methylundecane	26.34	1173	1173	MS, RI	Alkanes	7.96 ± 0.70a	4.91 ± 2.98b	2.88 ± 0.87b	0.23 ± 0.01c	0.27 ± 0.06c	0.31 ± 0.20c	0.14 ± 0.03c	0.94	0
65	Dodecane	27.3	1199	1200	MS, RI	Alkanes	13.32 ± 0.24a	8.87 ± 6.47ab	6.09 ± 2.11b	0.19 ± 0.02c	0.20 ± 0.06c	0.41 ± 0.29c	0.06 ± 0.00c	0.92	0
92	Tetradecane	33.26	1398	1400	MS, RI	Alkanes	5.23 ± 0.51ab	4.33 ± 1.99ab	2.92 ± 0.82b	7.00 ± 0.01a	6.94 ± 0.05a	4.75 ± 3.77ab	7.02 ± 0.03a	0.80	0.052
101	3-Methylpentadecane	37.66	1572	1574	MS, RI	Alkanes	1.67 ± 0.26a	1.61 ± 0.46a	1.08 ± 0.21b	0.17 ± 0.02c	0.13 ± 0.02c	0.12 ± 0.06c	0.08 ± 0.01c	0.87	0
104	Hexadecane	38.35	1599	1600	MS, RI	Alkanes	1.16 ± 0.08ab	1.25 ± 0.27a	0.96 ± 0.19b	0.22 ± 0.02c	0.17 ± 0.01c	0.12 ± 0.04c	0.08 ± 0.01c	0.87	0

18	1,3,5,7-Cyclooctatetraene	16.23	887	880	MS, RI	Alkenes	0.60 ± 0.08a	0.70 ± 0.09a	0.40 ± 0.07b	0.23 ± 0.02c	0.24 ± 0.05c	0.16 ± 0.04c	0.19 ± 0.05c	0.85	0
27	*α*-Phellandrene	20.03	993	1005	MS, RI	Alkenes	88.11 ± 6.13b	130.62 ± 29.86a	73.10 ± 13.87b	36.42 ± 4.74c	38.41 ± 7.11c	30.78 ± 7.67c	28.00 ± 4.41c	0.86	0
28	*β*-Myrcene	20.03	1002	1007	MS, RI	Alkenes	88.16 ± 6.11b	130.90 ± 29.53a	73.13 ± 13.92b	36.41 ± 4.75c	38.48 ± 7.17c	30.78 ± 7.67c	28.02 ± 4.41c	0.86	0
32	*δ*-Carene	21.11	1024	1017	MS, RI	Alkenes	2.62 ± 0.22b	3.29 ± 0.55a	1.85 ± 0.37c	0.76 ± 0.11d	0.83 ± 0.16d	0.63 ± 0.17d	0.53 ± 0.09d	0.85	0
36	*D*-Limonene	21.57	1037	1038	MS, RI	Alkenes	16.94 ± 0.76b	20.27 ± 3.84a	10.56 ± 2.13c	4.61 ± 0.62d	4.94 ± 0.99d	3.74 ± 1.05d	3.01 ± 0.55d	0.85	0
40	*β*-Ocimene	22.13	1053	1053	MS, RI	Alkenes	29.72 ± 3.09b	40.40 ± 6.50a	24.50 ± 4.64b	11.58 ± 1.50c	12.72 ± 2.39c	9.62 ± 2.59c	9.10 ± 1.25c	0.85	0
42	*β*-Terpinene	22.64	1068	1056	MS, RI	Alkenes	11.98 ± 18.23bc	40.39 ± 6.49a	24.50 ± 4.64b	11.57 ± 1.50bc	12.71 ± 2.38bc	9.61 ± 2.59c	9.10 ± 1.25c	0.96	0.002
52	(E, E)-2,6-dimethyl-1,3,5,7-octatetraene	24.81	1129	1130	MS, RI	Alkenes	0.77 ± 0.04b	0.92 ± 0.10a	0.59 ± 0.11c	0.25 ± 0.03d	0.26 ± 0.05d	0.21 ± 0.06d	0.21 ± 0.03d	0.85	0
53	(E, Z)-2,6-dimethylocta-2,4,6-triene	25.02	1135	1131	MS, RI	Alkenes	8.74 ± 0.45b	12.03 ± 2.77a	6.41 ± 1.21c	3.00 ± 0.40d	3.10 ± 0.53d	2.37 ± 0.58d	2.03 ± 0.38d	0.85	0
54	1,3,8-p-Menthatriene	25.15	1139	1127	MS, RI	Alkenes	2.67 ± 0.11b	3.40 ± 0.51a	2.16 ± 0.38c	0.97 ± 0.12d	1.02 ± 0.19d	0.80 ± 0.20d	0.78 ± 0.13d	0.84	0
63	1-Dodecene	27.05	1193	1192	MS, RI	Alkenes	0.93 ± 0.05b	0.94 ± 0.39b	2.56 ± 1.53a	0.70 ± 0.03b	0.61 ± 0.10b	0.51 ± 0.17b	0.67 ± 0.35b	1.14	0.015
69	(E)-3-Dodecene	27.46	1204	1185	MS, RI	Alkenes	0.22 ± 0.01a	0.20 ± 0.10ab	0.13 ± 0.03abc	0.02 ± 0.00d	0.11 ± 0.08bcd	0.02 ± 0.01cd	0.06 ± 0.07cd	0.89	0.003
78	4,5-Dimethyl-2,6-octadiene	29.3	1266	/	MS, RI	Alkenes	383.24 ± 98.38bc	608.81 ± 163.21a	428.08 ± 76.63b	257.80 ±	237.34 ± 8.84cd	233.26 ± 69.05cd	187.08 ± 10.14d	0.87	0

										25.96cd					
88	*α*-Cubebene	32.26	1365	1360	MS, RI	Alkenes	0.58 ± 0.25b	0.83 ± 0.12a	0.88 ± 0.17a	0.38 ± 0.02b	0.43 ± 0.13b	0.36 ± 0.15b	0.32 ± 0.03b	0.95	0.001
95	Caryophyllene	34.45	1446	1438	MS, RI	Alkenes	0.45 ± 0.05bc	1.15 ± 0.62a	0.85 ± 0.15ab	0.07 ± 0.01c	0.66 ± 0.13b	0.06 ± 0.04c	0.11 ± 0.02c	0.91	0
98	(Z)-Calamenene	37.02	1547	1546	MS, RI	Alkenes	4.02 ± 0.57a	4.19 ± 0.76a	3.50 ± 0.62a	1.13 ± 0.06b	1.18 ± 0.31b	0.99 ± 0.42b	1.26 ± 0.17b	0.89	0
100	*β*-Calacorene	37.57	1568	1566	MS, RI	Alkenes	0.76 ± 0.16a	0.77 ± 0.19a	0.64 ± 0.11a	0.21 ± 0.01b	0.23 ± 0.06b	0.20 ± 0.10b	0.38 ± 0.06b	0.94	0
105	Cadalene	40.74	1670	1674	MS, RI	Alkenes	0.38 ± 0.06b	0.57 ± 0.07a	0.37 ± 0.05b	0.13 ± 0.01c	0.13 ± 0.02c	0.09 ± 0.03c	0.13 ± 0.03c	0.88	0
80	Caprolactam	29.53	1273	1266	MS, RI	Amines	0.16 ± 0.02b	0.57 ± 0.52a	0.23 ± 0.04ab	0.12 ± 0.04b	0.25 ± 0.05ab	0.19 ± 0.03ab	0.11 ± 0.12b	0.95	0.169
9	Toluene	11.76	744	760	MS, RI	Aromatic hydrocarbons	1.17 ± 0.22b	1.75 ± 0.08a	0.98 ± 0.22b	0.42 ± 0.09c	0.57 ± 0.16c	0.62 ± 0.12c	0.60 ± 0.08c	0.91	0
16	1,3-Dimethylbenzene(m-xylene)	15.43	861	866	MS, RI	Aromatic hydrocarbons	0.92 ± 0.05a	1.00 ± 0.06a	0.52 ± 0.12b	0.24 ± 0.03c	0.25 ± 0.05c	0.19 ± 0.06c	0.17 ± 0.03c	0.86	0
34	*o*-Cymene	21.4	1032	1039	MS, RI	Aromatic hydrocarbons	14.66 ± 0.53a	16.27 ± 3.83a	7.63 ± 1.65b	3.21 ± 0.39c	3.15 ± 0.55c	2.62 ± 0.63c	1.94 ± 0.39c	0.86	0
47	1,2-Dimethyl-4-vinylbenzene	23.79	1100	1100	MS, RI	Aromatic hydrocarbons	3.54 ± 0.41a	4.14 ± 0.93a	2.20 ± 0.46b	0.94 ± 0.10c	0.93 ± 0.16c	0.71 ± 0.19c	0.57 ± 0.08c	0.84	0

41	(Z)-3-Hexenyl crotonate	22.36	1060	/	MS, RI	Esters	0.41 ± 0.08b	0.61 ± 0.10a	0.37 ± 0.07b	0.22 ± 0.03c	0.25 ± 0.04c	0.16 ± 0.05c	0.14 ± 0.01c	0.89	0
43	Formic acid, octyl ester	22.93	1076	1109	MS, RI	Esters	2.78 ± 0.36a	2.53 ± 0.12a	1.20 ± 0.23b	0.53 ± 0.08c	0.58 ± 0.12c	0.53 ± 0.17c	0.49 ± 0.11c	0.90	0
62	(E)-3-Hexenyl butanoate	26.9	1189	1185	MS, RI	Esters	23.62 ± 4.13b	30.98 ± 5.30b	43.82 ± 12.78a	15.38 ± 1.28cd	12.61 ± 3.51d	7.88 ± 3.34d	7.13 ± 1.16d	1.09	0
64	Octanoic acid, ethyl ester	27.19	1197	1197	MS, RI	Esters	1.10 ± 0.22a	1.11 ± 0.32a	1.35 ± 0.25a	0.18 ± 0.02b	0.20 ± 0.04b	0.16 ± 0.04b	0.18 ± 0.04b	0.97	0
67	(E)-2-Hexenyl butanoate	27.3	1200	1195	MS, RI	Esters	8.92 ± 0.21a	1.77 ± 0.59c	4.03 ± 2.22b	1.30 ± 0.05c	1.00 ± 0.31c	0.64 ± 0.28c	0.65 ± 0.25c	1.22	0
70	Methyl salicylate	27.55	1207	1206	MS, RI	Esters	126.18 ± 5.75b	218.76 ± 4.46a	220.82 ± 46.80a	93.92 ± 7.76bc	119.58 ± 24.33b	61.99 ± 24.16c	53.49 ± 5.73c	1.00	0
73	Bis(2-ethylhexyl) carbonate	27.75	1214	/	MS, RI	Esters	0.46 ± 0.07a	0.25 ± 0.14b	0.20 ± 0.06b	0.13 ± 0.03bc	0.13 ± 0.04bc	0.06 ± 0.07c	0.02 ± 0.00c	1.08	0
76	(Z)-3-Hexenyl pentanoate	28.36	1234	1235	MS, RI	Esters	4.58 ± 0.58b	7.62 ± 1.01b	23.12 ± 6.17a	7.83 ± 0.47b	6.82 ± 1.84b	5.76 ± 2.46b	6.90 ± 1.04b	1.30	0
82	Ethyl salicylate	29.88	1285	1286	MS, RI	Esters	0.47 ± 0.15c	1.47 ± 0.72b	3.63 ± 0.25a	0.40 ± 0.05c	0.63 ± 0.15c	0.15 ± 0.04c	0.13 ± 0.05c	1.18	0
83	3,7-Dimethyl-cis-2,6-octadienyl formate	30.56	1308	1282	MS, RI	Esters	1.36 ± 0.28b	5.74 ± 4.64a	3.21 ± 0.89ab	2.83 ± 0.05ab	3.70 ± 0.87ab	1.13 ± 0.52b	1.12 ± 0.16b	1.02	0.067
85	(E)-Methyl 3,7-dimethylocta-2,6-dienoate	31.19	1329	1321	MS, RI	Esters	5.98 ± 0.94c	12.29 ± 1.39b	18.51 ± 3.98a	6.52 ± 0.21c	5.83 ± 1.33c	3.45 ± 1.20c	4.51 ± 0.59c	1.13	0
86	Dimethyl salicylate	31.71	1346	1347	MS, RI	Esters	0.20 ± 0.06ab	0.17 ± 0.10b	0.34 ± 0.18a	0.15 ± 0.05b	0.17 ± 0.04b	0.09 ± 0.04b	0.10 ± 0.01b	1.16	0.052
90	(Z)-3-Hexenyl hexanoate	32.77	1382	1381	MS, RI	Esters	13.89 ± 4.99b	16.29 ± 4.67b	50.99 ± 13.58a	19.55 ± 1.61b	16.99 ± 4.45b	11.55 ± 5.84b	15.82 ± 5.15b	1.30	0

91	(Z)-3-Hexenyl (Z)-3-hexenoate	32.91	1387	1388	MS, RI	Esters	3.78 ± 0.58b	4.06 ± 0.48b	4.42 ± 1.12b	1.58 ± 0.18b	1.27 ± 0.30b	0.76 ± 0.32b	9.15 ± 6.42a	1.31	0.015
99	(R)-4,4,7a-Trimethyl-5,6,7,7a-tetrahydrobenzofuran-2(4H)-one (Dihydroactinidiolide)	37.42	1562	1548	MS, RI	Esters	0.80 ± 0.11bc	1.29 ± 0.32a	0.99 ± 0.17b	0.46 ± 0.04de	0.56 ± 0.14cd	0.25 ± 0.10e	0.26 ± 0.09e	0.91	0
103	(Z)-3-Hexenyl benzoate	38	1585	1575	MS, RI	Esters	0.11 ± 0.01c	0.29 ± 0.04c	1.30 ± 0.24a	1.14 ± 0.08a	1.26 ± 0.25a	0.80 ± 0.28b	0.81 ± 0.09b	1.15	0
6	2-Ethylfuran	9.49	671	684	MS, RI	Heterocyclic compounds	6.09 ± 2.25a	4.48 ± 0.39a	2.57 ± 0.81b	1.06 ± 0.02b	1.19 ± 0.24b	1.37 ± 0.47b	0.97 ± 0.08b	0.93	0
26	(E)-2-(1-Pentenyl)furan	20.03	993	1000	MS, RI	Heterocyclic compounds	9.65 ± 0.64ab	10.35 ± 8.72a	2.48 ± 3.40c	3.77 ± 0.50abc	4.00 ± 0.75abc	3.20 ± 0.82bc	2.03 ± 1.68c	0.94	0.06
38	Pyrazine	21.72	1041	747	MS, RI	Heterocyclic compounds	2.07 ± 0.63c	4.12 ± 0.88a	3.08 ± 0.51b	1.01 ± 0.08d	1.15 ± 0.24d	0.83 ± 0.23d	0.86 ± 0.12d	0.91	0
45	Linalool oxide	23.17	1083	1092	MS, RI	Heterocyclic compounds	9.64 ± 0.49b	21.20 ± 5.19a	11.39 ± 1.94b	5.17 ± 0.91c	5.44 ± 1.20c	4.48 ± 1.31c	3.30 ± 0.22c	0.93	0
61	*cis*-Linalool oxide (pyranoid)	26.67	1182	1183	MS, RI	Heterocyclic compounds	5.29 ± 0.26b	9.18 ± 2.23a	6.81 ± 1.50b	1.95 ± 0.24c	2.52 ± 0.62c	2.21 ± 0.67c	2.19 ± 0.21c	0.90	0
77	Quinoline	29.04	1257	1242	MS, RI	Heterocyclic compounds	0.26 ± 0.02b	0.35 ± 0.03a	0.27 ± 0.06b	0.11 ± 0.01c	0.11 ± 0.02c	0.08 ± 0.03c	0.07 ± 0.01c	0.86	0

84	Indole	30.61	1310	1303	MS, RI	Heterocyclic compounds	10.86 ± 1.66a	1.33 ± 0.92b	0.24 ± 0.03b	0.10 ± 0.02b	0.09 ± 0.02b	0.05 ± 0.02b	0.03 ± 0.00b	1.17	0
106	Caffeine	44.43	1788	1784	MS, RI	Heterocyclic compounds	0.61 ± 0.08b	1.43 ± 0.75ab	1.42 ± 0.25ab	1.24 ± 0.11ab	1.57 ± 0.59a	1.19 ± 0.65ab	0.70 ± 0.12ab	1.16	0.13
8	3-Penten-2-one	10.84	714	714	MS, RI	Ketones	0.40 ± 0.06a	0.38 ± 0.08a	0.17 ± 0.05b	0.07 ± 0.01c	0.11 ± 0.02bc	0.11 ± 0.02bc	0.07 ± 0.01c	0.89	0
17	2-Heptanone	16.11	883	889	MS, RI	Ketones	1.01 ± 0.13b	2.09 ± 0.51a	0.61 ± 0.17c	0.18 ± 0.03d	0.26 ± 0.05cd	0.36 ± 0.09cd	0.36 ± 0.05cd	1.02	0
20	5-Hexen-3-one	16.43	893	/	MS, RI	Ketones	0.08 ± 0.04b	0.26 ± 0.22a	0.03 ± 0.01b	0.02 ± 0.00b	0.03 ± 0.01b	0.02 ± 0.01b	0.01 ± 0.00b	0.97	0.032
25	6-Methyl-5-hepten-2-one	19.82	996	988	MS, RI	Ketones	1.82 ± 0.28b	2.80 ± 0.62a	0.89 ± 0.21c	0.38 ± 0.03d	0.42 ± 0.08cd	0.25 ± 0.07d	0.24 ± 0.01d	0.90	0
44	3,5-Octadien-2-one	22.97	1077	1090	MS, RI	Ketones	13.30 ± 1.78b	21.30 ± 0.36a	14.64 ± 3.53b	6.67 ± 0.11c	11.32 ± 2.23b	5.22 ± 1.86c	1.81 ± 0.18d	0.99	0
46	3-Nonanone	23.5	1092	1089	MS, RI	Ketones	0.17 ± 0.02b	0.23 ± 0.02a	0.13 ± 0.04c	0.03 ± 0.00de	0.03 ± 0.01de	0.06 ± 0.02d	0.02 ± 0.00e	0.85	0
50	2,3,4-Trimethyl-2-cyclopenten-1-one	24.17	1111	1089	MS, RI	Ketones	5.31 ± 7.83a	10.31 ± 15.66a	0.86 ± 0.19a	0.30 ± 0.01a	0.35 ± 0.07a	1.80 ± 2.79a	0.27 ± 0.04a	0.79	0.487
57	5-Ethyl-6-methyl-3(E)-hepten-2-one	25.66	1153	1143	MS, RI	Ketones	1.04 ± 0.10b	2.15 ± 0.52a	1.02 ± 0.24b	0.25 ± 0.04c	0.33 ± 0.10c	0.33 ± 0.12c	0.29 ± 0.07c	0.92	0
93	(Z)-Jasmone	33.61	1413	1405	MS, RI	Ketones	5.61 ± 0.97a	3.68 ± 0.77b	2.01 ± 0.18c	0.93 ± 0.04d	0.98 ± 0.19d	0.56 ± 0.20d	0.53 ± 0.08d	0.98	0
94	α-Ionone	34.37	1443	1438	MS, RI	Ketones	1.19 ± 0.23bc	2.07 ± 0.41a	1.34 ± 0.27b	0.67 ± 0.04de	0.82 ± 0.16cd	0.36 ± 0.12e	0.31 ± 0.05e	0.91	0

96	Geranyl acetone	34.8	1460	1453	MS, RI	Ketones	0.43 ± 0.04b	0.74 ± 0.02a	0.62 ± 0.11a	0.40 ± 0.01b	0.44 ± 0.10b	0.15 ± 0.05c	0.14 ± 0.11c	1.06	0
97	*β*-Ionone	35.93	1504	1505	MS, RI	Ketones	8.95 ± 1.31bc	17.60 ± 2.78a	11.37 ± 2.29b	6.31 ± 0.18cd	7.80 ± 1.64c	4.10 ± 1.60d	3.80 ± 0.77d	0.94	0
3	Acetic acid	7.22	599	600	MS, RI	Organic acids	0.82 ± 0.08a	0.76 ± 0.26a	0.66 ± 0.08a	0.13 ± 0.02b	0.06 ± 0.09b	0.15 ± 0.04b	0.19 ± 0.05b	0.94	0
87	Geranic acid	31.9	1353	1355	MS, RI	Organic acids	1.36 ± 0.07b	2.25 ± 0.61b	10.15 ± 3.26a	2.69 ± 1.06b	3.57 ± 0.82b	1.66 ± 0.90b	2.05 ± 0.55b	1.28	0
1	Dimethyl sulfide	5.77	552	550	MS, RI	Sulfur compounds	1.56 ± 0.40c	13.26 ± 1.28a	12.74 ± 3.00a	5.37 ± 0.85b	6.60 ± 1.23b	4.85 ± 1.60b	2.22 ± 0.23c	1.11	0

Data were expressed as mean ± SD (n = 3).

RT[a] represents retention time; RI[b] represents the linear retention indices calculated from a series of *n*-alkanes (C7-C40); RI[c] represents retention indices referred to the literature value with HP-5ms column or equivalent chromatographic column [NIST Chemistry WebBook (http://webbook.nist.gov/chemistry/) and http://www.flavornet.org/flavornet.html/]. ID represents identification method. MS represents identification based on the NIST 11 database. VIP represents variable importance in the projection. Values in a row followed by different letters are significantly different ($p < 0.05$).

Table S4 The screened volatile components with rOAVs greater than one in sweet and floral aroma black tea during the entire manufacturing process.

Compounds	Threshold values (μg/L)	Contents (μg/L)							rOAVs						
		Fresh leaves	Withering	Shaking	Rolling	Fermentation	First drying	Final firing	Fresh leaves	Withering	Shaking	Rolling	Fermentation	First drying	Final firing
Dimethyl sulfide	0.3	1.56	13.26	12.74	5.37	6.60	4.85	2.22	5.20	44.19	42.47	17.91	21.99	16.16	7.40
2-Methylpropanal	0.49	0.08	0.76	1.23	0.44	0.64	0.90	0.72	0.16	1.55	2.51	0.89	1.30	1.84	1.46
3-Methylbutanal	0.2	0.12	0.99	1.86	1.08	1.79	1.84	1.49	0.58	4.93	9.30	5.38	8.95	9.20	7.43
2-Methylbutanal	1	0.25	3.43	5.93	2.06	3.06	4.81	4.34	0.25	3.43	5.93	2.06	3.06	4.81	4.34
2-Ethylfuran	2.3	6.09	4.48	2.57	1.06	1.19	1.37	0.97	2.65	1.95	1.12	0.46	0.52	0.60	0.42
Hexanal	4.5	2.49	7.96	3.63	4.08	7.94	6.82	2.30	0.55	1.77	0.81	0.91	1.77	1.52	0.51
(E)-2-Hexenal	17	16.43	4.39	8.02	16.11	19.60	6.94	3.90	0.97	0.26	0.47	0.95	1.15	0.41	0.23
1-Hexanol	5.6	6.85	5.11	3.99	1.91	1.82	1.31	0.93	1.22	0.91	0.71	0.34	0.32	0.23	0.17
2-Heptanone	1	1.01	2.09	0.61	0.18	0.26	0.36	0.36	1.01	2.09	0.61	0.18	0.26	0.36	0.36
1-Octen-3-ol	1	7.36	12.51	4.60	1.93	2.31	1.96	1.28	7.36	12.51	4.60	1.93	2.31	1.96	1.28
α-Phellandrene	36	88.11	130.62	73.10	36.42	38.41	30.78	28.00	2.45	3.63	2.03	1.01	1.07	0.86	0.78
β-Myrcene	15	88.16	130.90	73.13	36.41	38.48	30.78	28.02	5.88	8.73	4.88	2.43	2.57	2.05	1.87
o-Cymene	11.4	14.66	16.27	7.63	3.21	3.15	2.62	1.94	1.29	1.43	0.67	0.28	0.28	0.23	0.17
β-Ocimene	0.02	29.72	40.40	24.50	11.58	12.72	9.62	9.10	1485.87	2020.19	1225.20	578.85	635.82	480.95	455.01
3,5-Octadien-2-one	0.5	13.30	21.30	14.64	6.67	11.32	5.22	1.81	26.59	42.60	29.28	13.35	22.64	10.44	3.63
Linalool	0.22	141.23	210.87	109.38	47.01	45.98	37.29	31.93	641.97	958.49	497.16	213.70	209.02	169.52	145.13
Decanal	5	13.37	8.49	6.08	0.33	0.20	0.21	4.07	2.67	1.70	1.22	0.07	0.04	0.04	0.81
Methyl salicylate	40	126.18	218.76	220.82	93.92	119.58	61.99	53.49	3.15	5.47	5.52	2.35	2.99	1.55	1.34
β-Cyclocitral	3	69.21	121.19	125.74	52.26	67.02	33.52	28.75	23.07	40.40	41.91	17.42	22.34	11.17	9.58

β-Citral	53	383.03	607.54	427.69	257.35	291.93	232.73	186.95	7.23	11.46	8.07	4.86	5.51	4.39	3.53
Citral	30	282.82	685.14	26.26	17.29	19.82	106.03	66.47	9.43	22.84	0.88	0.58	0.66	3.53	2.22
β-Ionone	0.01	8.95	17.60	11.37	6.31	7.80	4.10	3.80	895.11	1759.80	1136.86	631.48	780.28	409.87	379.56

rOAVs represents the relative odor activity values of volatile compounds.

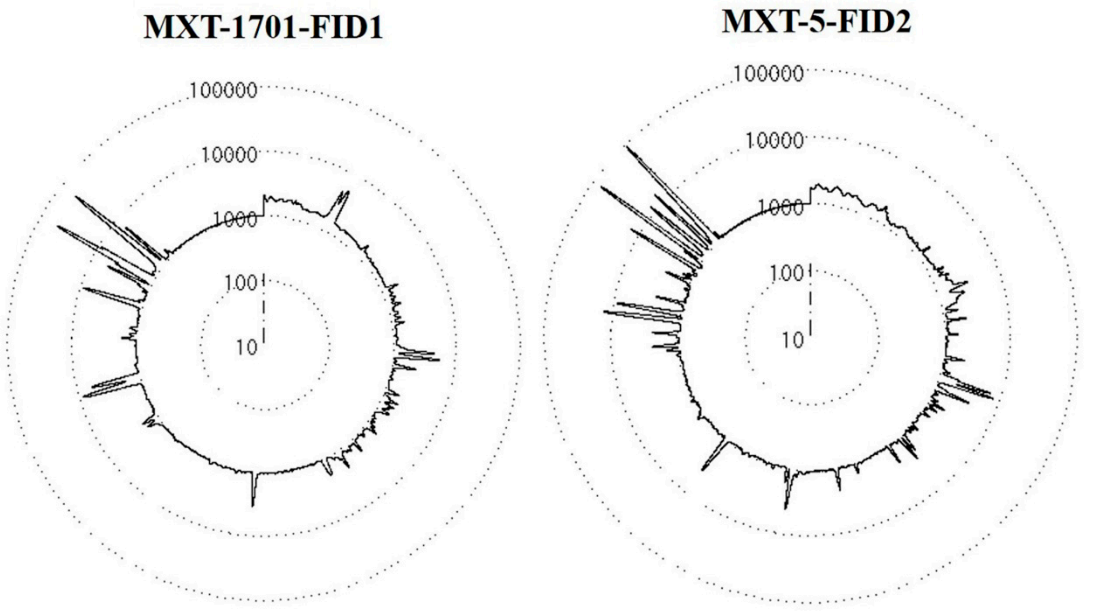

Fig. S1 Radar map of sweet and floral aroma black tea in final firing stage by using GC-E-Nose.

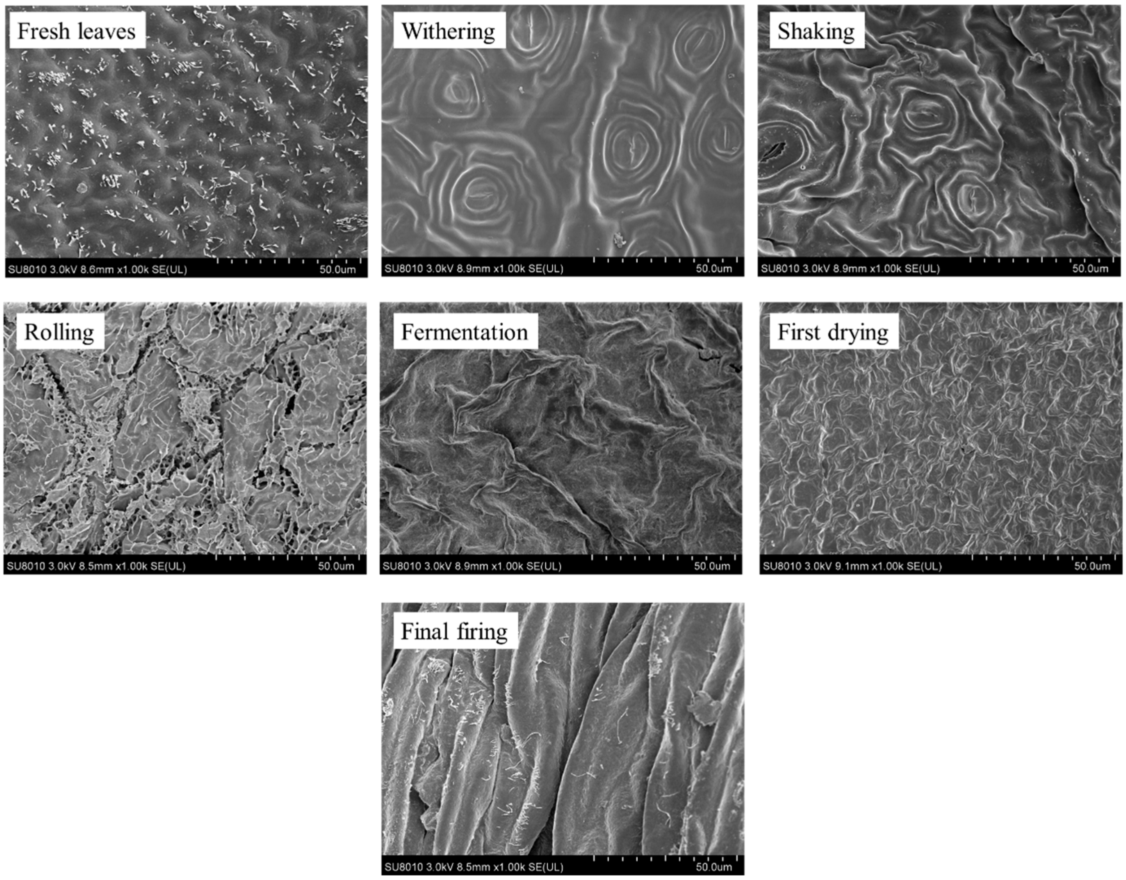

Fig. S2 The microstructures of sweet and floral aroma black tea during the manufacturing process obtained from scanning electron microscopy.
